# Mechanical stress caused by wind on leaves of *Theobroma cacao*: Photosynthetic, molecular, antioxidative and ultrastructural responses

**DOI:** 10.1371/journal.pone.0198274

**Published:** 2018-06-27

**Authors:** Graciele Santos Monteiro Reis, Alex-Alan Furtado de Almeida, Pedro Antônio Oliveira Mangabeira, Ivanildes Conceição dos Santos, Carlos Priminho Pirovani, Dário Ahnert

**Affiliations:** Department of Biological Sciences, State University of Santa Cruz, Campus Soane Nazaré de Andrade, Rodovia Jorge Amado, Ilhéus, BA, Brazil; Institute for Sustainable Plant Protection, C.N.R., ITALY

## Abstract

*Theobroma cacao* is cultivated in the shade, in a so-called 'Cabruca' system, in intercropped with *Erithryna* or other tree species of economic value, and in full sun as a monoculture in irrigated or chemically-irrigated systems. Since it is a species quite intolerant to wind, it is practically impossible to implant cacao crops under full exposure to the sun, or in areas of frequent winds, without the protection of windbreaks, using arboreal species around the area of culture in the form of box. Wind can cause mechanical stimuli in plants, affecting their growth and development. The objective of this work was to evaluate the photosynthetic changes in mature leaves and the molecular, biochemical and ultrastructural changes in young and mature leaves of the CCN 51 cloned genotype of *T*. *cacao* subjected to intermittent (IW) and constant (CW) wind, with velocities of 2.5, 3.5 and 4.5 m s^-1^, during 3, 6 and 12 h of exposure. It was verified that CW and IW, considering different exposure times, interfered directly in stomatal conductance (*gs*), transpiration (*E*) and water use efficiency (*WUE*), causing a reduction of the photosynthetic rate (*A*) in mature leaves. In addition, the pulvinus and blade of young and mature leaves, exposed to IW and CW with different exposure times (3 and 12 h), showed marked macroscopic and microscopic mechanical injuries resulting from the constant leaf movement. At both speeds, there was rupture of the cell nuclear membrane in pulvinus and the mesophyll tissues, mainly in the young leaves. On the other hand, in young and mature leaves exposed to CW and IW at different speeds and exposure times, there was lipid peroxidation, increased activity of guaiacol (GPX) and ascorbate (APX) peroxidases in most treatments; and altered expression of transcripts of *psba* and *psbo* genes related to the phothosynthetic apparatus and *Cu-Zn-sod* and *per* genes related to antioxidative enzymes at the rate of 4.5 m s^-1^. Younger leaves were more intolerant to mechanical stress caused by the wind, since presented greater macro and microscopic damages and, consequently, greater molecular, biochemical and ultrastructural changes. High wind speeds can seriously compromise the development of young leaves of *T*. *cacao* plants and affect their productivity.

## Introduction

Mechanical stimuli caused by various environmental factors such as wind, touch, rainfall and obstacles [1] may interfere with plant growth and development [2]. Wind action on the plants can cause several mechanical damages, such as alterations in the cell walls [3], premature falling of leaves, breaking of branches and even uprooting of the entire plant, consequently generating a series of physiological effects and changes in response to water loss, hormonal changes and reduction in the rate of cellular stretching, among others [4–6]. Wind action may also promote decreases in plant vitality, with consequent reductions in productivity [7].

Responses to the wind may vary between different parts of the plant and across plant species. The leaves are the most intolerant organs for this type of stress, the effects of which directly affect photosynthesis and transpiration [8,9]. Depending on leaf characteristics and wind speed, in some cases partial reduction or total elimination of the boundary air layer on the leaf surface [6,10] were observed. Partial reduction usually occurs in conditions of weak or moderate wind, consequently increasing the gaseous exchanges at leaf level; total elimination is usually verified in the presence of strong wind, which promotes the closure of the stomata. Increased transpiration reduces leaf temperature and can dehydrate the plants [11]. Therefore, the effects of wind can increase [12] or reduce the photosynthetic rate, reducing CO_2_ diffusion resistance [13] or lowering leaf temperature below the optimum level, and promoting stomatal closure, to avoid excessive water loss [8,9,13–15].

Reactive oxygen species (ROS) play a key role in the process of acclimatization of plants to abiotic stress [16]. ROS are partially reduced or activated forms of atmospheric oxygen, such as superoxide (O_2_^-^), hydrogen peroxide (H_2_O_2_) and hydroxyl (OH), and are considered as unavoidable by-products of aerobic metabolism. However, ROS excess, when not eliminated by normal cell antioxidative systems, can exert a wide range of physiological responses in plants, such as oxidative damage to membranes (lipid peroxidation), proteins, RNA molecules and DNA; it can trigger oxidative stress, programmed cell death, and alter gene expression [17]. In light of this, ROS are considered toxic by-products of the stress metabolism but they also function as signal transduction molecules, which regulate different pathways during plant acclimatization to stress [5,6,16]. However, the plants have developed an efficient mechanism to protect themselves from ROS toxicity by means of enzymatic and non-enzymatic cell detoxification [18–21]. In terms of enzymatic means, it is worth mentioning the activity of superoxide dismutase (SOD), ascorbate peroxidase (APX), catalase (CAT), guaiacol peroxidase (GPX) [21]. Certain studies have demonstrated an increase in the tolerance to abiotic stress through the genetic manipulation of antioxidant enzymes responsible for the elimination of ROS [22–24].

The existing information on the effects of mechanical wind action on *T*. *cacao* plants is quite scarce. In areas of frequent wind, it has been observed that it is practically impossible to implant cacao plants without the protection of windbreaks [4]. Physiological, biochemical and molecular analyses may elucidate the mechanical changes promoted by the action of wind in cacao, in addition to contributing to the understanding of the mechanisms used by these plants to adapt and/or adjust to the mechanical stress. The objective of this study was to evaluate the photosynthetic, molecular, biochemical and ultrastructural changes in young and mature leaves of rooted cutting seedlings of CCN51 cultivar exposed to intermittent and constant winds during 3, 6 and 12h exposure periods.

## Material and methods

### Plant material and growing conditions

The experiment was conducted under greenhouse conditions to protect the cloned saplings against the wind. The CCN51 seedlings were obtained by rooted cuttings of plagiotropic branches of plants with 10 years old grown plagiotropic branches of CCN 51 cacao plants, at the *Biofábrica de Cacau* Institute. After rooting (four months of age), the cloned plants were grown in 16 L plastic pots containing soil.

During the experimental period, we monitored the photosynthetically active radiation (PAR), using light radiation sensors S-LIA-M003 coupled with the climatological Hobo Micro Station Hobo Data Logger (Onset Computer, Massachusetts, USA) and the temperature (T) and air relative humidity (RH) inside the greenhouse, through sensors Hobo H8 Pro (Onset, Computer, Massachusetts, USA). The average values of PAR, T and RH were 974 ± 4 μmol photons m^-2^ s^-1^, 26 ± 3°C and 74 ± 2%, respectively.

### Wind tunnel

**Fig 1:**
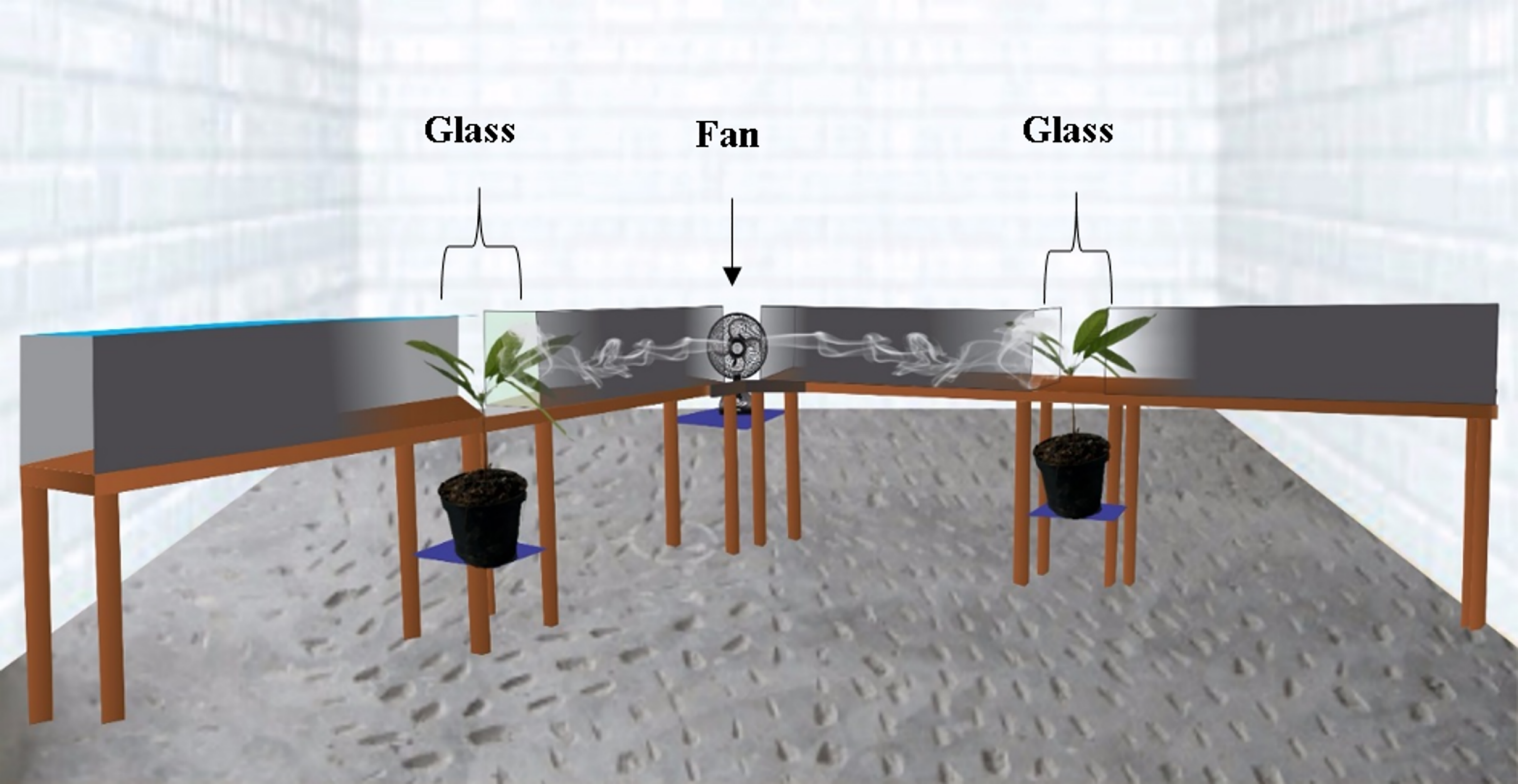
Schematic representation of the wind tunnel used in the experiment. A fan with a swinging axle (120°) provided intermittent and also constant (fixed-axis) air currents within the tunnels with velocities of 2.5 (S1), 3.5 (S2) and 4.5 (S3) m s^-1^. The tunnels were made of wood, at 90° angle, each extending for 4 m in length, with a cross section measuring 0.50 m wide x 0.40 m high, containing a transparent glass box of 0.50 m x 0.40 m in the centers of its longitudinal axes, provided with a hole in their lower part where the cloned plants were introduced.

At seven months of age, seedlings of the cloned genotype CCN51 were exposed to intermittent (IW) and constant (CW) winds in a wind tunnel (Fig 1) and kept inside the greenhouse in the absence of wind (control). Two wind tunnels, made of wood, were built at a 90° angle, each one being 4 m in length with a cross-section measuring 0.50 m wide x 0.40 m high. The tunnels were supported by wooden feet 1.0 m high and containing a transparent glass box 0.50 m x 0.40 m with a hole in the lower part and positioned in the centers of their longitudinal axes, where we have introduced the cloned plants. The pots were balanced over a wooden support just below the tunnel, allowing the aerial part of the seedlings to be exposed to the wind flow. A fan with a swinging axle (120°) provided intermittent and constant air currents with its fixed axis within the tunnels, with velocities of 2.5 (S1), 3.5 (S2) and 4.5 (S3) m s^-1^, measured by a mini-anemometer (CASSELA C 7748 / Z). The average wind velocity was recorded and in the region it corresponds to 2 m s^-1^. Six cloned plants were examined per treatment, exposed for 3 (T1), 6 (T2) and 12 (T3) h to the intermittent and constant air flows, together with the control treatment (no wind and exposure time).

### Leaf gas exchange

Instantaneous measurements of leaf gas exchange were performed at 3, 6 and 12 h after exposure of the cloned plants to the intermittent (IW) and constant (CW) wind flows, on a completely expanded and mature leaf of each plant per treatment, by means of a portable system for photosynthesis measurements LI-6400 (Li-Cor, USA), equipped with a 6400-02B RedBlue artificial light source. During the measurements, the assimilation chamber temperature was set at 26°C, the photosynthetically active radiation was maintained at 700 μmol m^-2^ s^-1^, above saturation irradiance for *T*. *cacao* (without occurrence of photoinhibition) and Atmospheric CO_2_ concentration of 380 μmol mol^-1^ by means of the CO_2_ injection system of the apparatus, methodology used by [25]. The minimum time for stabilization of each reading was 120 s, and the maximum time for saving each reading of 150 s. The maximum coefficient of variation for each reading was 0.3%. The rates of net photosynthesis (*A*) and transpiration (*E*) per unit of leaf area and stomatal conductance to water vapor (*gs*) were estimated from the values of CO_2_ variation and humidity inside the chamber determined by infrared gas analyzer of the apparatus. In addition to these parameters, the CO_2_ molar fraction in the intercellular space of the mesophyll (*Ci*) and the instantaneous (*WUE*) and intrinsic (*A/gs*) efficiencies of water use, and vapor pressure deficit (*VPDL*) were also calculated.

### Ultrastructural analysis

**Fig 2:**
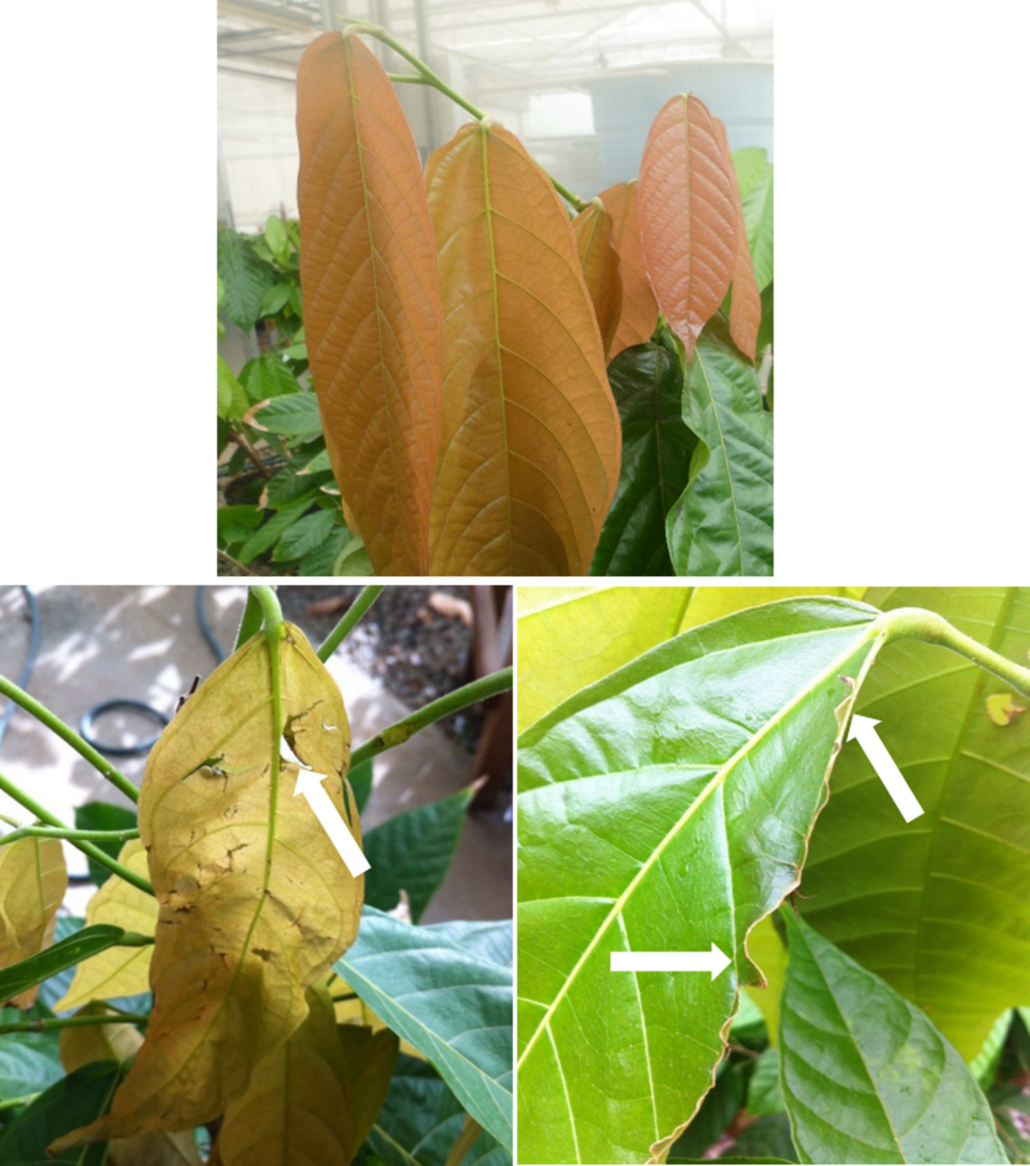
Young leaves of cloned plants of the CCN 51 genotype of *T*. *cacao* exposed to constant wind, with a velocity of 4.5m s^-1^ for a period of 12 h. Young leaves shortly after being exposed to the wind (A). Young leaves before exposure to wind (B) and 10 days after exposure to wind (C). Black arrow—Damage caused by wind.

Immediately after exposure of cloned plants of CCN51 to IW and CW in wind tunnels (Fig 2), we performed the collection of pulvinus and limbus from young and mature leaves. For ultrastructural analysis in transmission electron microscopy (MET), samples of plants that were exposed to wind for 12 h (T3), with wind speed of 2.5 (S1) and 4.5 (S3) m s^-1^, were collected to assess the damages caused by the mechanical stress promoted by the wind. Samples were prefixed in 3% glutaraldehyde, in a sodium cacodylate buffer at 0.1 M, pH 6.8, during 4 hours. Subsequently, sections of pulvinus and leaf limbs were fixed in osmium tetroxide (OsO_4_) in the same buffer for 1.0 h, dehydrated in acetone series and included in resin. Ultra-thin sections were obtained using the Reichert-Jung microtome and analyzed in the MET Morgani-268-D.

### Programmed cell death (PCD)

For the detection of PCD, pulvinus of young leaves exposed them for 12 h to CW with velocity of 4.5 m s^-1^ were collected. The samples were fixed in formaldehyde, transferred to 70% alcohol and kept at 4°C in a refrigerator. The plant material was embedded in paraffin and subsequently cut by a the rotating microtome. The sections obtained were dewaxed with xylol, dehydrated in ethanolic series, made water-proof and exposed to the TUNEL reaction (Terminal deoxinucleotidyl transferase Uracil Nick End Labeling) using the *In situ Cell Death Detection Kit* (AP) (Boehringer Mannheim; Tunnel Assay), according to the manufacturer's recommendations. The TUNEL probes were observed on a Leica DM RXA2 fluorescence microscope equipped with a Leica MPS 60 digital camera and the images captured using the I3 filter (450–490 nm excitation).

### Antioxidative enzymes

After exposure of young and mature leaves to IW and CW in the wind tunnel for 3, 6 and 12 h, pulvinus and leaves were collected, frozen in liquid nitrogen and lyophilized for analysis of enzymatic activity. The activities of guaiacol peroxidase (GPX, EC 1.11.1.7) was obtained according to the protocol described by [26], with modifications. For the enzymatic assay, we used 96-well Microplates containing 140 μL of reaction buffer POD 2 x, 40 mmoles L^-1^ of guaiacol, 0.06% (v/v) H_2_O_2_ and sodium phosphate (20 mmoles L^-1^, pH 6.0), 139 μL of phosphate buffer (50 mmoles L^-1^, pH 6.0), and 1 μL of enzyme extract previously diluted. The reading was conducted in a microplate spectrophotometer (VERSAmax). The guaiacol peroxidase activity was expressed with the increase in consumption of guaiacol in μmol h^-1^ g^-1^ of dry matter. The conversion of the data was obtained in absorbance values at 470 nm min^-1^ g^-1^ of dry matter for the consumption of guaiacol in mmol h^-1^ g^-1^ of dry matter. Ascorbate peroxidase (APX, EC 1.11.1.11) were determined according to the methodologies described by [27], with modifications. In the reaction, the presence of APX in the vegetable extract reduces the H_2_O_2_ concentration of the medium, as a function of the reduction of ascorbic acid added. To the diluted plant extract were added to the reaction buffer (50 mmoles L^-1^ potassium phosphate, 0.5 mmoles L^-1^ ascorbate, 0.1 mmoles L^-1^ EDTA and 0.1 mmoles L^-1^ H_2_O_2_). The reaction will start with the addition of ascorbate. The decay will be monitored at the wavelength of 290 nm for 300 s, with readings every 30 s. The activity will be determined in Spectramax Paradigm microparticle spectrophotometer (Molecular Devices). The analysis will be performed in quadruplicates and the values expressed in μmol ascorbate g^-1^ DW min^-1^.

### Lipid peroxidation

Lipid peroxidation of cell membranes, evaluated by thiobarbituric acid reactive substances (TBARS), was performed according to the protocol described by our colleagues [28]. Young and mature leaves were collected after exposure of CCN51 plants to IW and CW in a wind tunnel for 3, 6 and 12 hours. Approximately 0.02 g of lyophilized samples were ground in 0.1% trichloroacetic acid (TCA) and homogenized in 2 mL of 0.1% aqueous TCA solution. The extracts were then centrifuged for 6 min. at 10,000 x g at 4°C. From the supernatant were pipetted 0.5 mL of extract into tubes for the reaction. In the reaction tubes were then added 1.5 mL of 0.5% thiobarbituric acid in 20% TCA. 1.5 mL of 20% TCA was added to the blank tubes. Soon after, the tubes were kept in a water bath at 95°C for 30 minutes. Then, the reactions were immediately stopped on ice. After cooling, the contents were centrifuged for 6 minutes at 10,000 x g. Finally, the accumulated TBARS concentration was determined by reading the absorbance of the reactions at 532 nm.

### Gene expression

Young and mature leaf blades of clonal plants exposed for 3, 6 and 12 hours at highest wind velocity (S3) were evaluated for gene expression at the transcript level. Immediately after collection, the samples were placed in liquid nitrogen and stored in ultra-freezer at -80°C and then lyophilized. The RNA was extracted with RNAqueous kit (Ambion). Then, the purity and integrity of the RNA were tested by 1% agarose gel electrophoresis. RNA samples were used for cDNA synthesis using Revertaid H-Minus Reverse Transcriptase (Fermentas), according to the manufacturer's instructions. Reactions were incubated at 65°C for 5 min, 37°C for 5 min, 42°C for 60 min and 70°C for another 10 min.

Relative quantitative real-time PCR (qPCR) was performed on a "Real Time PCR" thermocycler (*Applied Biosystems*, model 7500) using non-specific detection sequence (fluorophore), *SYBR Green I* (Roche). The abundance of transcripts was analyzed by means of specific primers (Table 1) of the genes *psba*, *psbo*, *per* and *Cu-Zn-sod* of the chloroplast and the cytoplasm drawn from the analysis of the known gene sequences of the *T*. *cacao* library (*http*:*//cocoagendb*.*cirad*.*fr*). In order to test the quality of these primers, the specificity and identity of the reverse transcription products, the qPCR products were monitored after each PCR by a reaction product analysis curve, able to distinguish gene-specific from nonspecific PCR products.

**Table 1:** Pairs gene-specific primers that were used in the analysis of qRT-PCR.

Gene	Accession numbers	Related proteins	Primer
PsbA	NC_014676.2 ^b^	D1 protein	F; 5’-GGTTTGCACTTTTACCCGA-3’
			R; 5’- CTCATAAGGACCGCCATT -3’
PsbO	CL326Contig1^a^	PsbO protein	F 5’-GCAAACGCTGAAGGAGTT-3’
			R 5’-GGCTTGAAGGCAAATGAGTC-3’
Per-1	CK144296.1^c^	Peroxidase class III	F 5’-TGCAACCATGAGTGGTGTCA- 3’
			R; 5’-CAGACGAGGGAAAGGAATGA- 3’
Cu-Zn-sod	CL94Conting1^a^	Cytosolic Cu-Zn SOD	F; 5’-GATGATGGCTGTGTGAGTTTCTCT- 3’
			R; 5’-CAACAACAGCTCTTCCAATAATTGA- 3’
Cu-Zn-sod	CL872Conting1^a^	Chloroplast Cu-Zn SOD	F; 5’-AATGGATGCATGTCAACAGGAGC- 3’
			R; 5’-GATGATGGCTGTGTGAGTTTCTCT- 3’

^a^http://esttik.cirad.fr/index.html

^b^http://www.ncbi.nlm.nih.gov/

^c^http://cocoagendb.cirad.fr/

The reaction mixture consisted of: cDNA template (500 ng), 0.5 μM of each primer, and 10 μL *SYBR Green* I fluorophore (Fermentas) in a final reaction volume of 20 μl. The temperature of the PCR products was raised from 55 to 99°C at a rate of 1°C/5s, and the resulting data was analyzed using the *LightCycler* software. Only a single band with a characteristic melting point was observed for each sample, indicating that qPCR produced a specific product from the used primers. Values for the *Threshold Cycle* (C_T_) were determined using the *LightCycler* software. Relative gene expression numbers were as a percentage of the control using the 2^-ΔΔCt^ method [29] and malate dehydrogenase (MDH) and glyceraldehyde 3-phosphate dehydrogenase (GAPDH) as endogenous control in order to detect changes in abundance of transcripts (Table 1). All reactions were prepared in triplicate and performed twice. For each treatment, three biological replicates were used for each evaluation.

### Multivariate analysis

The analysis of Principal Components was performed using the leaf gas exchange and the evaluated antioxidant metabolism variables obtained from plants subjected to different wind speeds. Initially, the gas exchange variables (*A*, *gs*, *E*, *Ci*, *VPDL* e *A/gs*), the antioxidant metabolism variables (TBARS, APX and GPX in leaves) were standardized, since they use different units.

### Statistical analysis

Four experiments were carried out in randomized blocks, in a 3x4 factorial scheme, each containing 12 treatments [3 wind speeds x 3 exposure times (+ control)] and 6 replications, to evaluate (1) mature leaves exposed to the intermittent wind; (2) mature leaves exposed to constant wind; (3) young leaves exposed to intermittent wind; And (4) young leaves exposed to constant wind. In the case of intermittent wind, two clonal plants were used per experimental unit. Analyses of variance (ANOVA) and comparisons between means of treatments using the Scott-knott test (p <0.05) were performed.

## Results

### Macroscopic and ultrastructural analysis of leaves

The leaves of the CCN51 cloned plants when exposed to constant wind (CW) with a velocity of 4.5 m s^-1^ for a period of 12 h showed ruptures and breaches around the edges and mesophyll. Also, the mesophyll showed dark coloration, probably due to the oxidation of phenols provoked by the mechanical stress generated by the wind action when exposing the interior of the leaf mesophyll (Fig 2). On the other hand, although the pulvinus and the blade experienced noticeable mechanical injuries resulting from constant leaf movement, there was no leaf fall.

**Fig 3:**
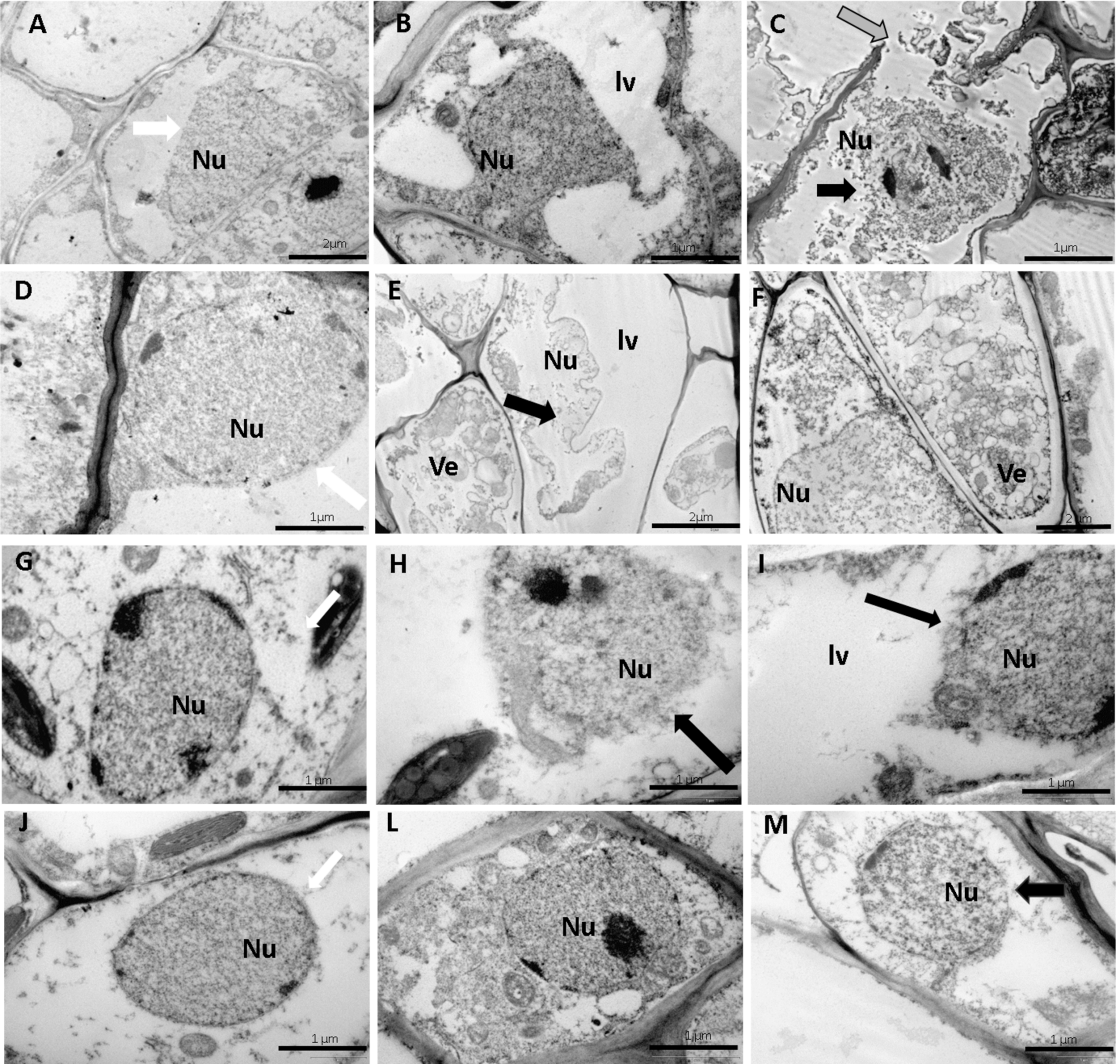
Ultrastructural micrographs of leaf pulvinus cells from CCN 51 cloned plants of *T*. *cacao*. Young leaf pulvinus: control (A and D), constant wind (B) and intermittent (E), with velocity of 4.5 m s^-1^ for, 3 h; (C) intermittent wind and constant wind (F) with a velocity of 4.5 m s^-1^ for 12 h. Mature leaf pulvinus: control (G and J); Constant (H) and intermittent (L) wind with velocity of 4.5 m s^-1^ for 3 h; constant wind (I) and intermittent (M) with velocity of 4.5 m s^-1^ for 12 h. Black arrow—Disruption of the nuclear membrane. White arrow—nuclear membrane intact. Gray arrow—cell wall rupture. Nu—nucleus. Ve—vesicles. Lithic vacuole. Bars: 1 and 2μm.

**Fig 4:**
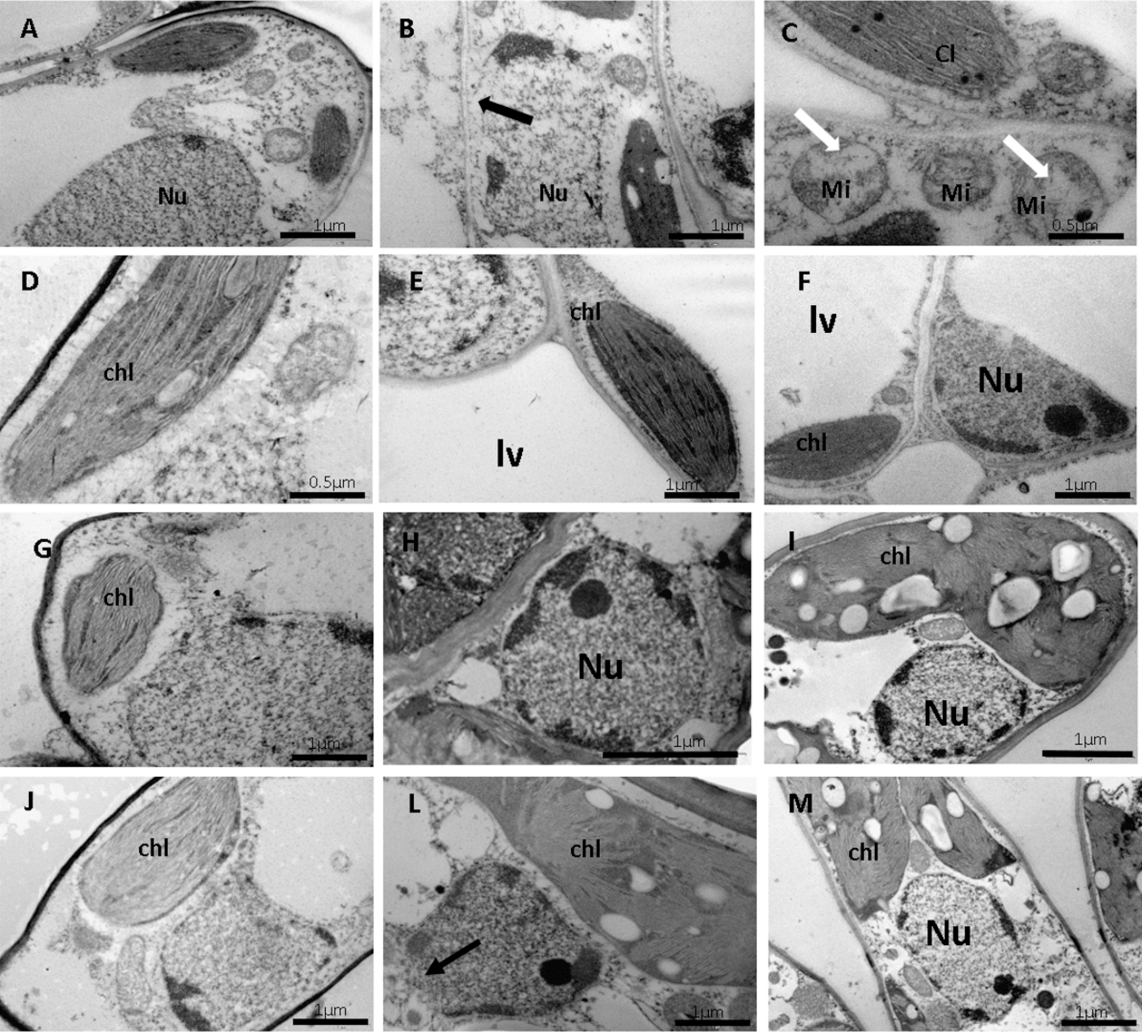
Ultrastructural micrographs of mesophyll cells from CCN 51 cloned plants of *T*. *cacao*. Young leaf: control (A and D), constant wind (B) and intermittent (E), with velocity of 4.5 m s^-1^ for, 3 h; (C) intermittent wind and constant wind (F) with a velocity of 4.5 m s^-1^ for 12 h. Mature leaf: control (G and J); constant (H) and intermittent (L) wind with velocity of 4.5 m s^-1^ for 3 h; Constant wind (I) and intermittent (M) with velocity of 4.5 m ^s-1^ for 12 h Black arrow—disintegration of cell membrane; White arrow–intact cell membrane. Gray arrow–cell membrane disruption; Nu–nucleus. Mi–mitochondria. Lv–lytic vacuole. Bars: 1 and 2 μm.

**Fig 5:**
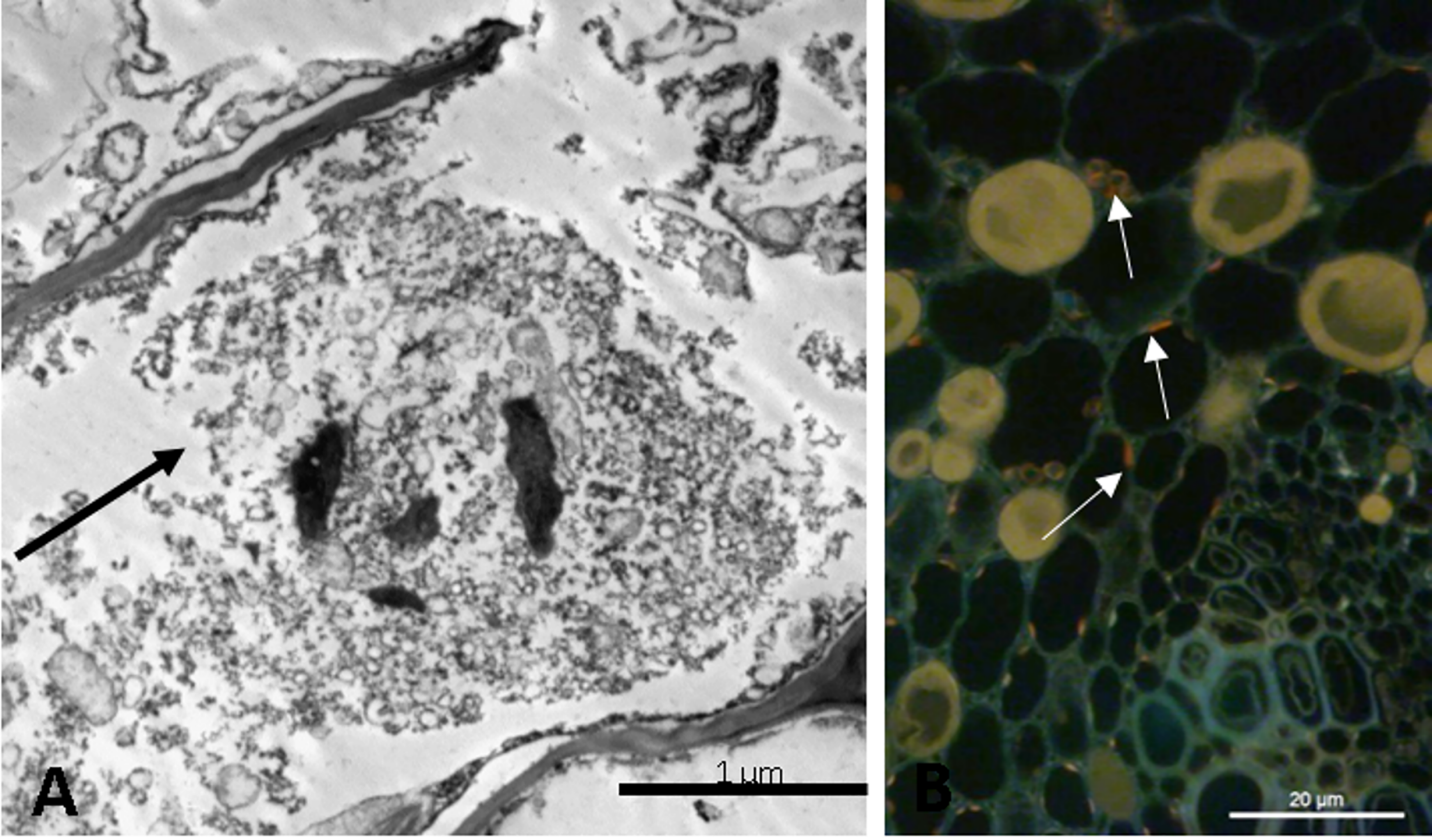
Typical programmed cell death (PCD) features. The figure shows some specific characteristics of the PCD observed in mesophyll cells of young leaves of *T*. *cacao*, exposed to constant wind for 12 h. (A) Ultrastructural micrograph evidencing the rupture of cell membrane. (B) Detection of DNA fragmentation by means of the *Tunnel* reaction. Bar = 1 μm and 20 μm.

The wind caused alterations in the cellular ultrastructures of the pulvinus (Fig 3A–3M) and leaf mesophyll (Fig 4A–4M), especially in young leaves when exposed to CW with a velocity of 4.5 m s^-1^ for a period of 12 h. There was rupture of the cell walls and nuclear membranes at the pulvinus level (Fig 3E and 3F), especially in young leaf pulvinus exposed to CW and IW, for a period of 3 and 12 h, respectively. This was also observed in mature leaves exposed to CW. In these same treatments, nuclear retraction (Fig 3E), vesicle formation (Fig 3E and 3F) and lytic vacuole (Fig 3B, 3E and 3I) were observed. PCD was detected in young leaf pulvinus by tunnel reaction (Fig 5B). In addition, there was damage to the mitochondria of mesophyll cells in young leaves exposed to CW for 3 and 12 h (Fig 4B and 4C) and lythic vacuole formation in young leaves exposed to IW for 3 and 12 h (Fig 4E and 4F).

### Leaf gas exchanges

**Fig 6:**
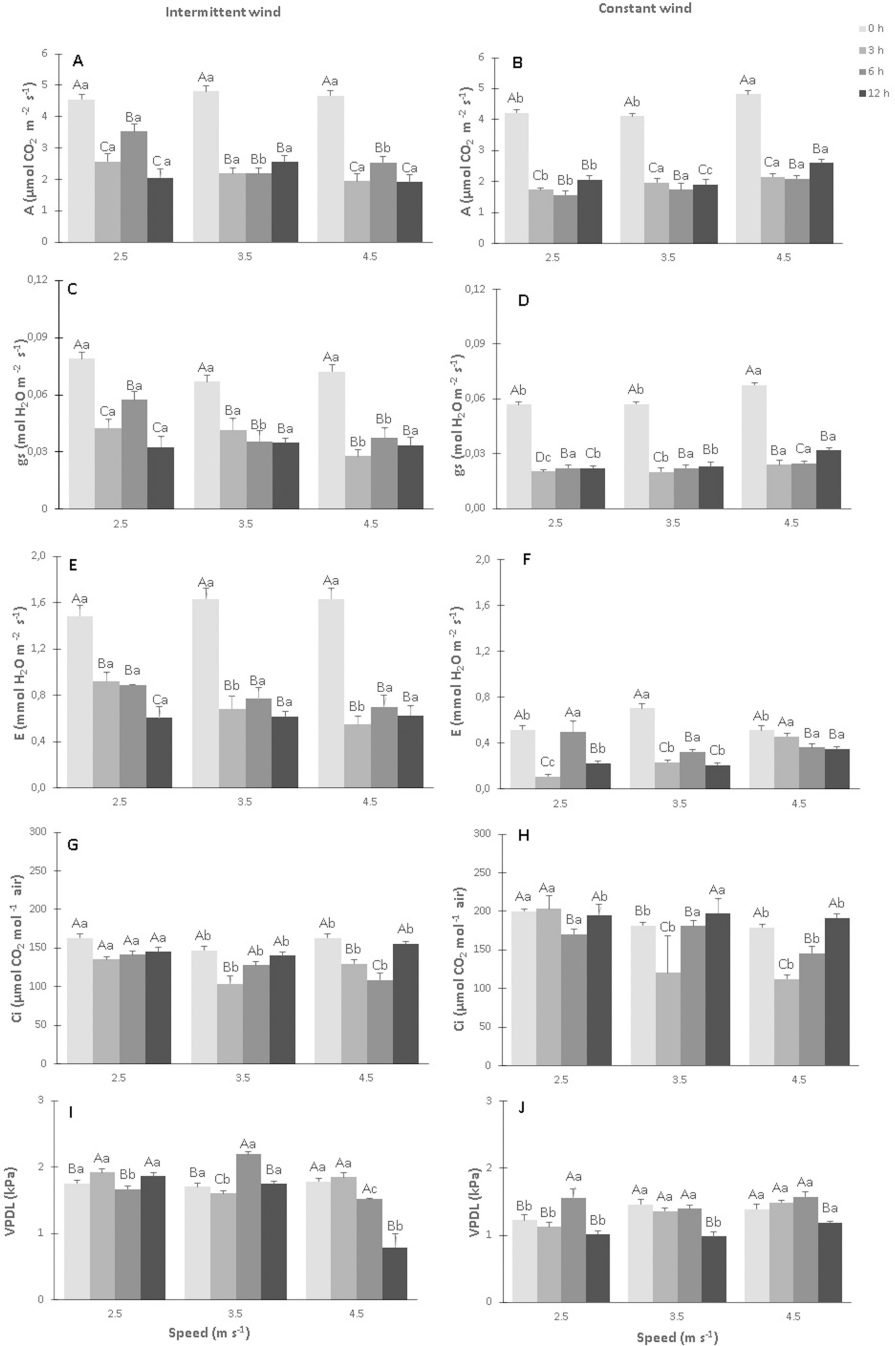
Changes in the net photosynthesis (A)–(A and B), stomatal conductance (*gs*)—(C and D), transpiration (*E*)—(E and F), internal CO_2_ concentration in leaf mesophyll (*Ci*)–(G and H) and water steam pressure deficit between leaf and air (*VPDL*)–(I and J) in CCN 51 cloned plants of *T*. *cacao* exposed to intermittent and constant wind at different speeds [2.5, 3.5 and 4.5 m s^-1^] and exposure times (0, 3, 6 and 12 h). Averages between exposure times and between speeds followed by the same uppercase and lowercase letters, respectively, do not differ by Scott-Knott test (p <0.05). Mean values of four replicates (± SE).

**Fig 7:**
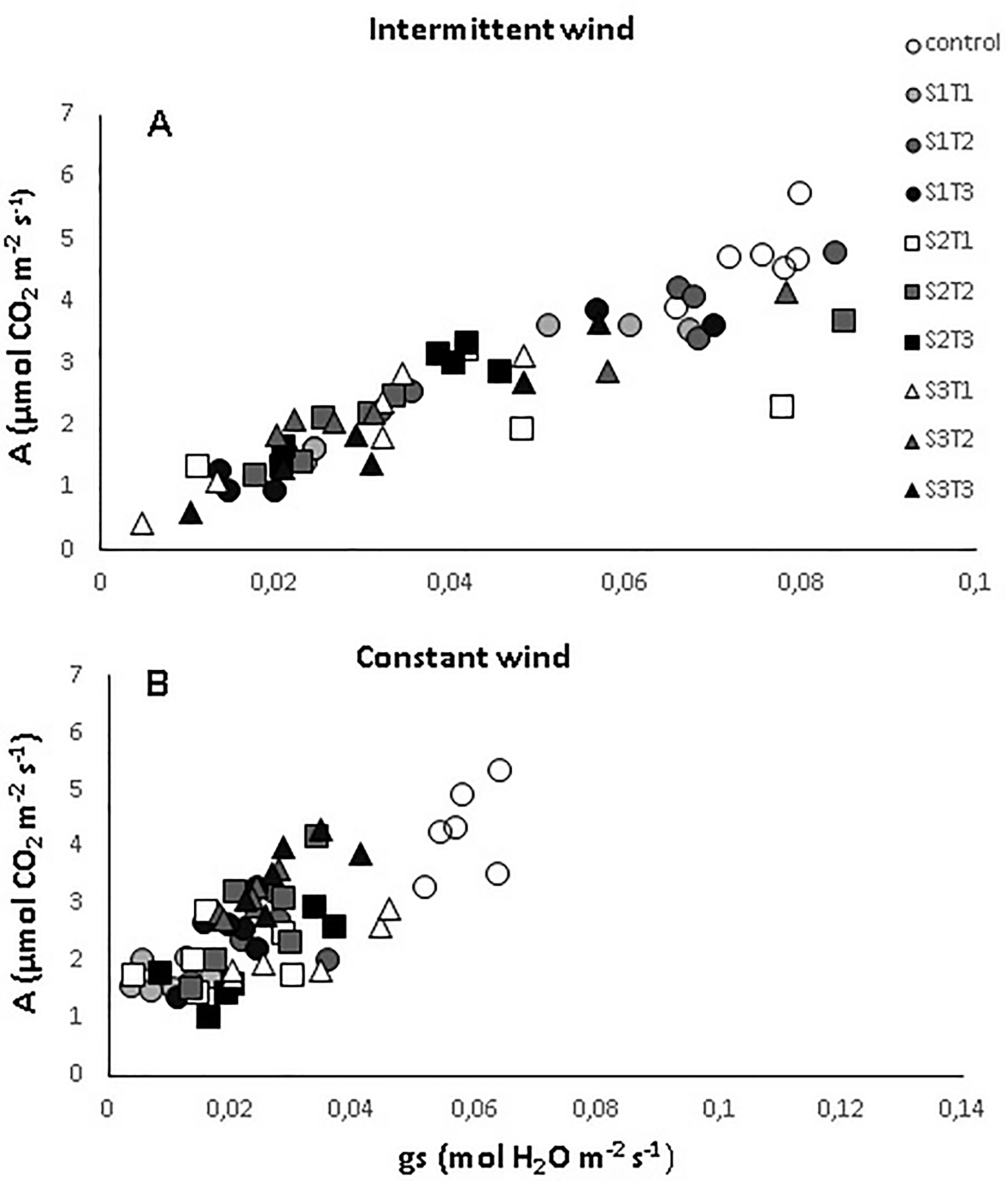
Relationship between net photosynthesis (*A*) and stomatal conductance (*gs*) in CCN 51 cloned plants of *T*. *cacao* exposed to intermittent and constant wind at different speeds [2.5 (S1), 3.5 (S2) and 4.5 (S3) m s^-1^] and exposure times [3 (T1), 6 (T2) and 12 (T3) h)–(S1T1, S1T2, S1T3, S2T1, S2T2, S2T3, S3T1, S3T2 and S3T3). Averages between exposure times and between velocities followed by the same uppercase and lowercase letters, respectively, do not differ by Scott-Knott test (p<0.05). Mean values of four replicates (± SE).

There was a decrease in net photosynthesis (*A*), stomatal conductance (*gs*) and transpiration (*E*) in the leaves of cacao plants, with increasing intermittent wind velocity (IW), and a small variation in gas exchange between the wind exposure times when compared to the control (Fig 6A, 6C and 6E, S1 Table). In the control plants, *A*, *gs* and *E* values of were 4.5 μmol CO_2_ m^-2^ s^-1^, 0.07 mol H_2_O m^-2^ s^-1^ and 1.6 mmol H_2_O m^-2^ s^-1^, respectively. The lowest values for *A*, *gs* and *E* in the plants exposed to IW were 1.92 μmol CO_2_ m^-2^ s^-1^ in S3T3, 0.02 mol H_2_O m^-2^ s^-1^ in S3T1 and 0.5 mmol H_2_O m^-2^ s^-1^ in S3T1, respectively. On the other hand, in the plants exposed to CW, values of *A*, *gs* and *E* were 4.2 μmol CO_2_ m^-2^ s^-1^, 0.05 mol H_2_O m^-2^ s^-1^ and 0.5 mmol H_2_O m^-2^ s^-1^, respectively, in comparison to the control plants (Fig 6B, 6D and 6F, S1 Table). In contrast, the lowest values of *A*, *gs* and *E* were found in the S1T2, S2T1 and S1T1, respectively, whose values were 1.5 μmol CO_2_ m^-2^ s^-1^, 0.01 mol H_2_O m^-2^ s^-1^ and 0.1 mmol H_2_O m^-2^ s^-1^, respectively. Moreover, and as expected, there was a direct proportional relationship between *A* and *gs* in leaves of the plants exposed to IW and CW (Fig 7A and 7B, S2 Table).

The values of Ci were reduced in plants exposed to IW, mainly at exposure times of 3 and 6 h at all wind speeds (Fig 6G, S1 Table). The lowest values (p<0.05) were 120.1 and 112.5 μmol CO_2_ mol^-1^ air for S2T1 and S3T1, respectively, in relation to control. Control Ci lowest value was 179.3 μmol CO_2_ mol^-1^ air. However, the plants exposed to CW (Fig 6H) presented varied values in the concentration of Ci. The highest value (p<0.05) of Ci was 236.7 μmol CO_2_ mol^-1^ air in S2T3, and the lowest value was 120.5 μmol for S3T1, while the control showed a value of 206.5 μmol CO_2_ mol^-1^ air.

Values of VPDL varied for IW and CW in relation to the respective controls (Fig 6I and 6J, S1 Table), whose values were 1.75and 1.3 kPa for IW and CW, respectively. The highest values (p<0.05) were 2.19 kPa for S2T2 and 1.55 kPa for S1T2, respectively for IW and CW, while the lowest values (p<0.05) were 0.79 kPa for S3T3 and 0.98 kPa for S2T3, respectively for IW and CW.

**Fig 8:**
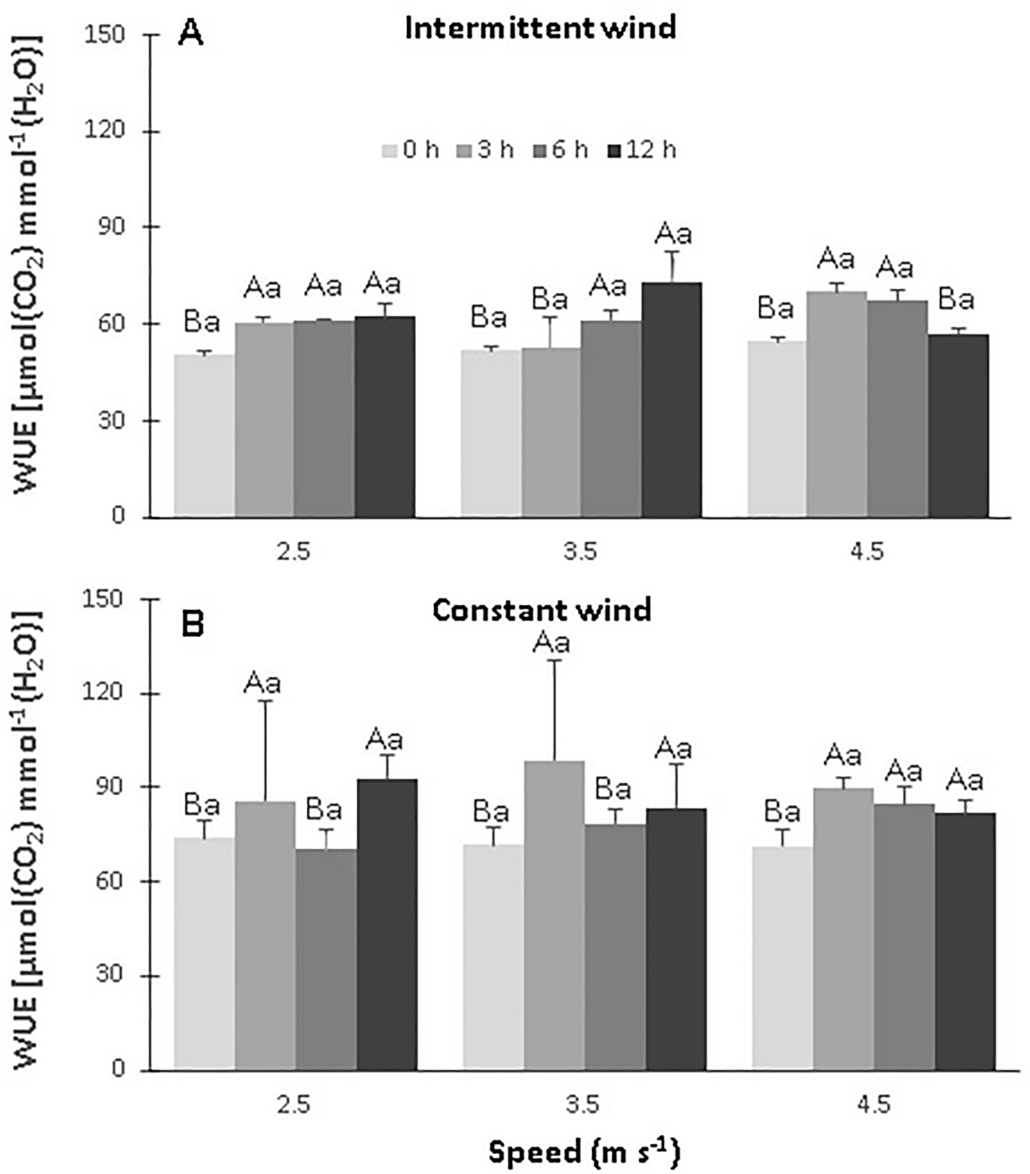
Instant water use efficiency (WUE) on leaves of CCN 51 cloned plants of *T*. *cacao* exposed to intermittent and constant winds with different speeds [2.5 (S1), 3.5 (S2) and 4.5 (S3) m s^-1^] and exposure times [0 (C), 3 (T1), 6 (T2) and 12 (T3) h). Averages between exposure times and between speed followed by the same uppercase and lowercase letters, respectively, do not differ by Scott-Knott test (p<0.05). Mean values of four replicates (± SE).

The instantaneous water use efficiency (WUE) increased in all treatments exposed to IW, especially for the combinations S2T3, S3T1 and S3T2, whose values were 73.3, 74.5, 70.2 and 67.2 μmol CO_2_ mmol H_2_O ^-1^. The highest value of the control was 54.5 μmol CO_2_ mmol H_2_O ^-1^ (Fig 8A, S3 Table). In plants exposed to CW there was a significant increase (p <0.05) in WUE in most treatments (Fig 8B). The highest values occurred in the combinations S1T3, S2T3, S3T1 whose values were 92.9, 98.6, 89.9 μmol of CO_2_ mmol H2O ^-1^, respectively, when compared to the mean value of the control of 72.3 μmol of CO2 mmol H2O^-1^.

### Lipid peroxidation

**Fig 9:**
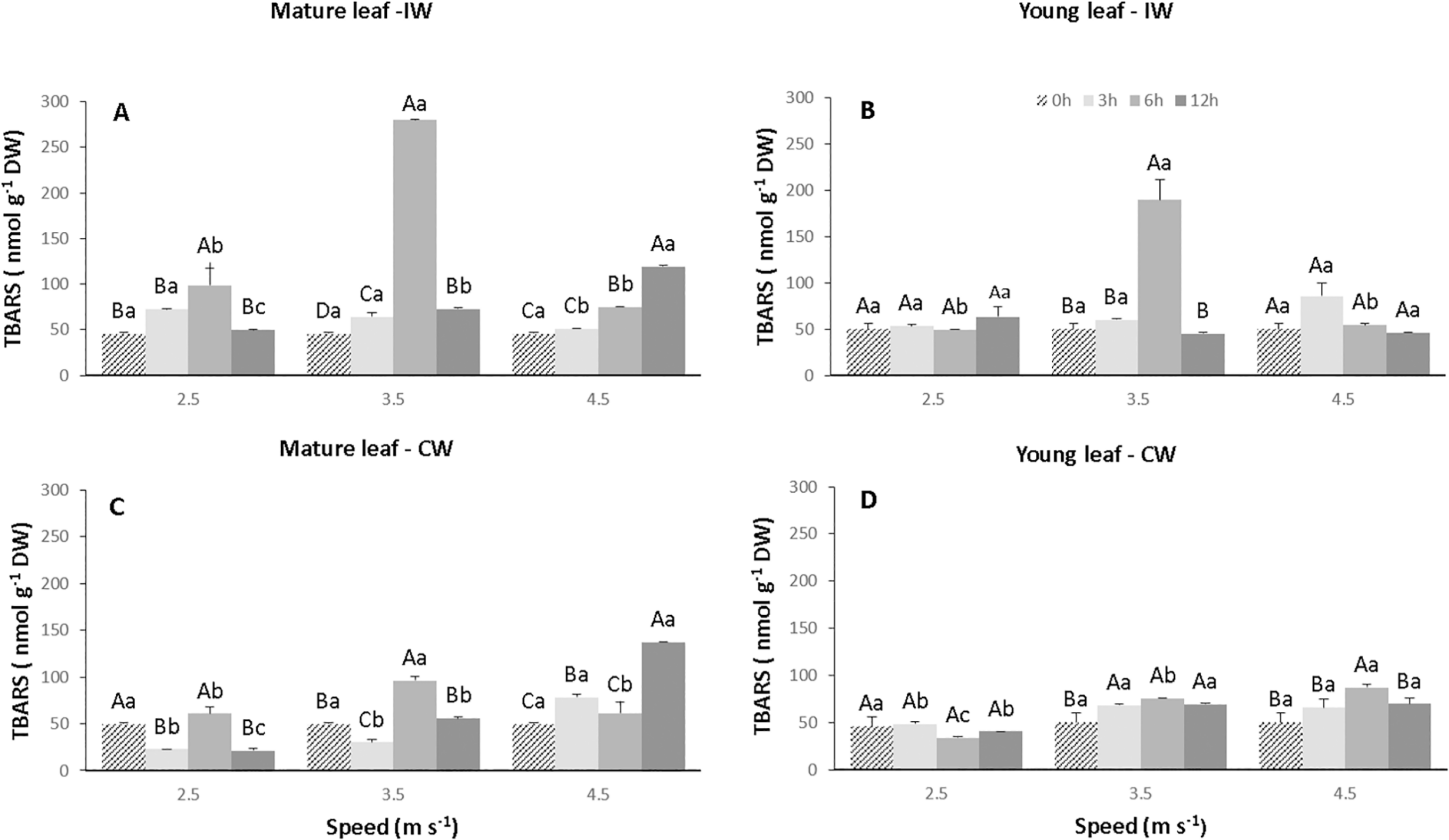
Concentration of thiobarbituric acid-reactive substances (TBARS) in leaves of CCN 51 cloned genotype of *T*. *cacao* exposed to intermittent (IW) and constant (CW) wind with different speeds (2.5, 3.5 and 4.5 m s^-1^) and exposure times (0, 3, 6 and 12 h). Mature and young leaves exposed to IW (A and B), respectively. Mature and young leaves exposed to CW (C and D), respectively. Averages between exposure times and between speeds followed by the same uppercase and lowercase letters, respectively, do not differ by Scott-Knott test (p<0.05). Mean values of four replicates (± SE).

In the young and mature leaves of the control plants, the accumulation of thiobarbituric acid reactive substances (TBARS) was 49.1 and 46.0 nmol g^-1^ DW, respectively (Fig 9A and 9B, S4 Table). In the young leaves of plants exposed to IW there was a significant increase (p<0.05) in the S2T2 combination of 189.2 nmol g^-1^ DW (Fig 9B), whereas in the mature leaves there was a significant increase (p<0.05) of TBARS, practically for all treatment combinations (Fig 9A). However, in plants exposed to CW, the highest values (p<0.05) for young leaves were 75.4 and 86.8 nmol g^-1^ DW for the combination S2T2 and S3T2, (Fig 9D) and 96.4 and 137.2 nmol g^-1^ DW for for mature leaves in the S2T2 and S3T3 combinations, respectively (Fig 9C).

### Enzymatic activity

**Fig 10:**
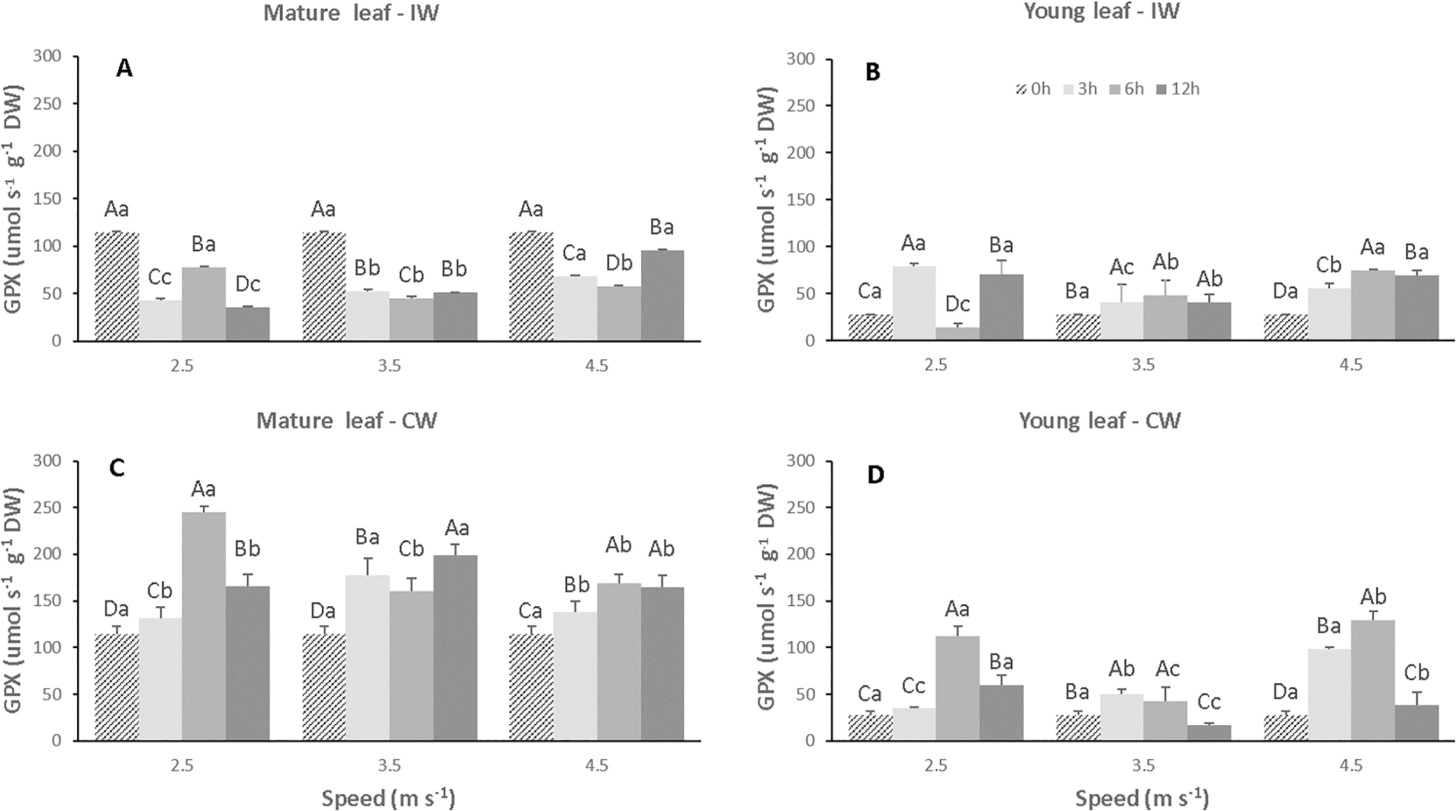
Activity of guaiacol peroxidase (GPX) in leaves of CCN 51 cloned plants of *T*. *cacao* exposed to wind at different speeds (2.5, 3.5 and 4.5 m s^-1^) and exposure times (0, 3, 6 and 12 h). Mature and young leaves exposed to intermittent wind (A and B), respectively. Mature and young leaves exposed to constant wind (C and D), respectively. Averages between exposure times and between velocities followed by the same uppercase and lowercase letters, respectively, do not differ by Scott-Knott test (p<0.05). Mean values of four replicates (± SE).

There was a decline in guaiacol peroxidase activity (GPX) in mature leaves of the CCN51 cloned plants exposed to IW in the three wind speeds (S1, S2 and S3) and in the three exposure times (T1, T2 and T3), when compared to the control (Fig 10A, S5 Table). In the control plants, mature leaves showed activity of 114.6 μmols^-1^ g^-1^ DW, whereas in mature leaves of the plants exposed to IW the activity of GPX ranged from 42.9 to 95.1 μmol s^-1^ g^-1^ DW (Fig 10A). In contrast, there was an increase in GPX activity in young leaves (Fig 10B). The highest values found for GPX in young leaves were 78.9, 47.9 and 75.3 μmol s^-1^ g^-1^ DW for treatments S1T1, S2T3, S3T2, respectively, whereas the control showed an activity of 27.6 μmol s^-1^ g^-1^ DW. On the other hand, mature leaves of CW plants showed a significant increase (p<0.05) in GPX activity in all treatments (Fig 10C), while young leaves exposed to S1T2 and S3T2 combinations showed activity of GPX of 112.0 and 129.7 μmol s^-1^ g^-1^ DW, respectively, which is larger than that for young leaves of the control sample, whose activity was 27.6 μmol s^-1^ g^-1^ DW (Fig 10D).

**Fig 11:**
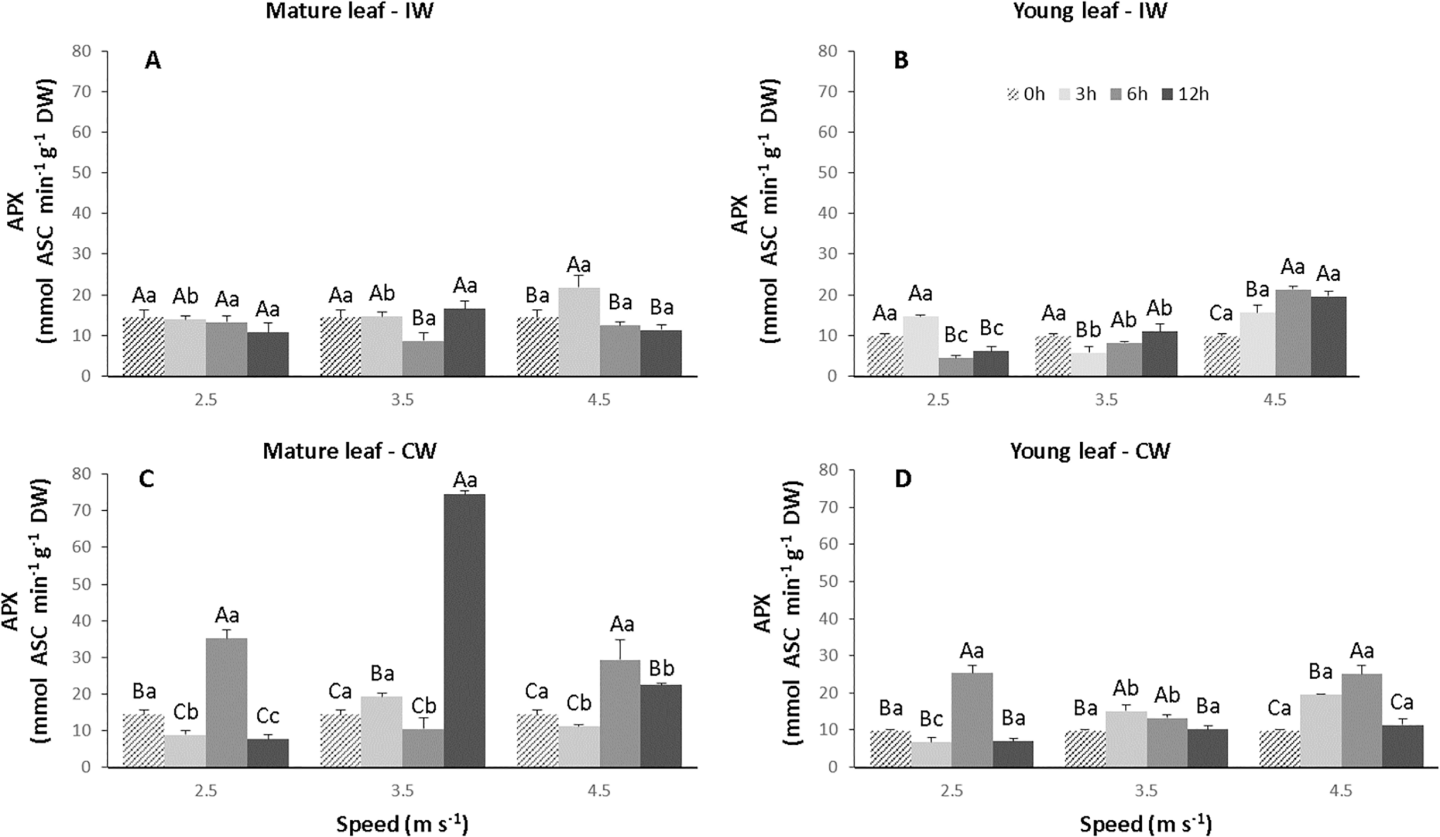
Activity of ascorbate peroxidase (APX) on leaves of CCN 51 cloned plants of *T*. *cacao* exposed to different wind speeds (2.5, 3.5 and 4.5 m s^-1^) and exposure times (0, 3, 6 and 12 h). Mature and young leaves exposed to intermittent wind (A and B), respectively. Mature and young leaves exposed to constant wind (C and D), respectively. Averages between exposure times and between speeds followed by the same uppercase and lowercase letters, respectively, do not differ by Scott-Knott test (p<0.05). Mean values of four replicates (± SE).

In regards to ascorbate peroxidase activity (APX) in the plants exposed to IW, when compared to the control, it was observed that in mature leaves there was a significant increase (p<0.05) from 14.3 to 21.7 μmol s^-1^ g^-1^ DW only the combination S3T1 (Fig 11A, S6 Table). In young leaves, there was a significant increase (p<0.05) in APX activity at the highest wind speed (4.5 m s^-1^) for all exposure times. The highest values of APX activity were 21.5 and 19.6 μmol s^-1^ g^-1^ DW, whereas for control it was 9.7 μmol s^-1^ g^-1^ DW (Fig 11B). On the other hand, the mature leaves of the plants exposed to CW presented greater APX activity, when compared to the control (14.3 μmol s^-1^ g^-1^ DW). In the combination S1T2, S2T3, S3T2 and S3T3 the values of APX activity were 35.10, 74.5, 29.35 and 22.46 μmol s^-1^ g^-1^ DW, respectively (Fig 11C). However, in young leaves, there was a significant increase (p<0.05) in APX activity of 25.3 and 25.0 μmol s^-1^ g^-1^ DW in the combination corresponding to S1T2 and S3T2, respectively (Fig 11D).

### Gene expression

**Fig 12:**
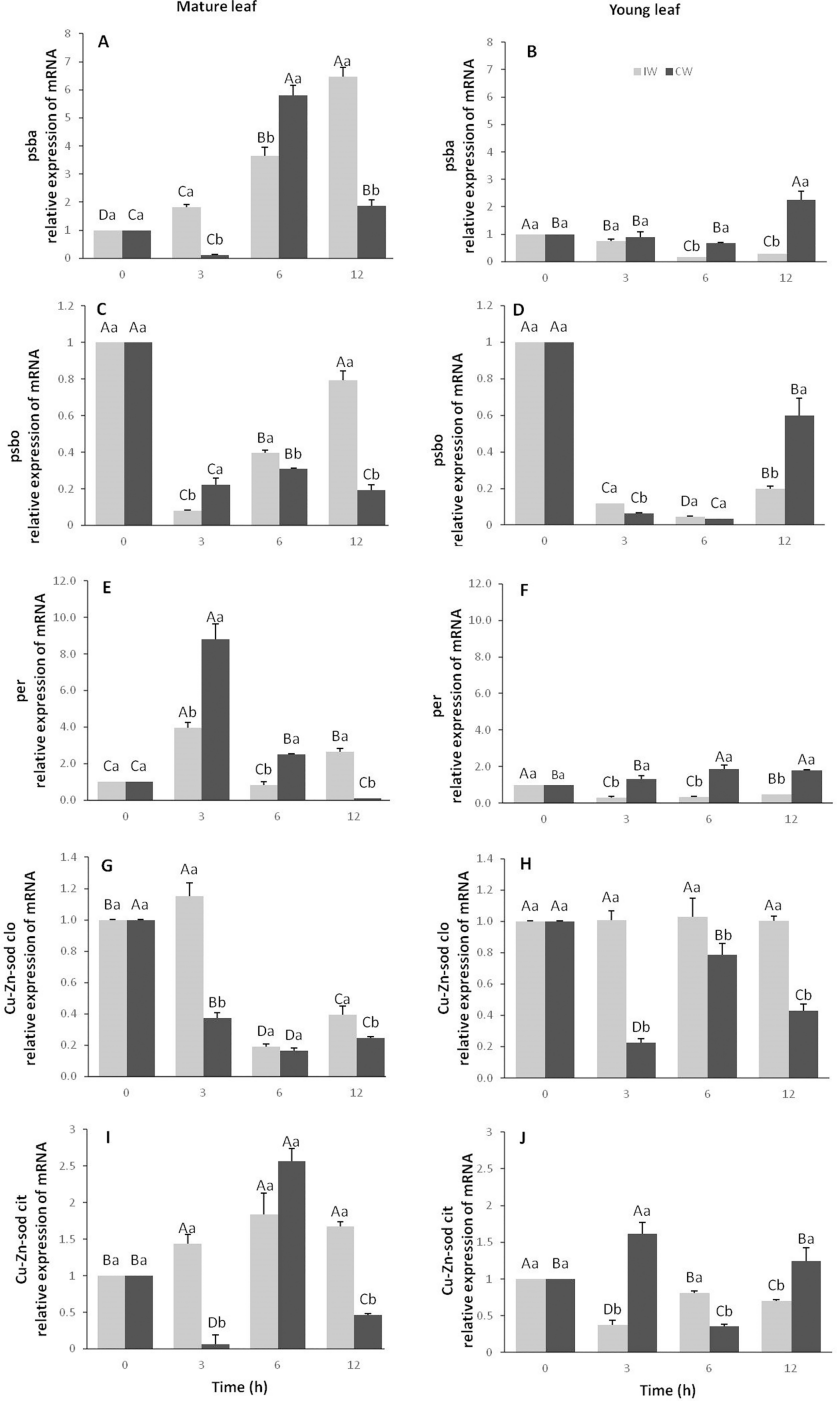
Relative expression of *psba*, *psbo*, *per*, *Cu-Zn-sod* in the chloroplast and cytoplasm genes in young and mature leaves of CCN 51 cloned plantas of *T*. *cacao* exposed to intermittent and constant wind. Genes *psba* (A and B), *psbo* (C and D), *per* (E and F), *Cu-Zn-sod* in the chloroplast (G and H) and in the cytoplasm (I and J). Averages between intermittent wind (IW) and constant wind (CW) and between exposure times followed by the same uppercase and lowercase letters, respectively, do not differ by Scott-Knott test (p<0.05). Mean values of nine replicates (± SE).

For the evaluation of *psba* and *psbo* gene expressions at the transcript level, young and mature leaves of the CCN51 cloned plants were exposed to constant (CW) and intermittent (IW) wind for periods of 3, 6 and 12h at the highest wind speed (S3). It was found a 6-fold increase in *psba* expression in mature leaves exposed to IW, parallel to time of exposure, when compared to the control (Fig 12A, S7 Table). However, when exposed to CW, mature leaves showed an increased *psba* expression of approximately 4.8-fold in T2 and 0.8-fold in T3 (Fig 12A). On the other hand, the young leaves presented a reduction in the expression of these transcripts in IW and CW (Fig 12B), except for T3, where there was a 2-fold increase in *psba* expression in relation to the control. In addition, there was a reduction in the expression of the *psbo* gene in the mature and young leaves of all treatments of IW and CW (Fig 12C and 12D, S7 Table).

The increase in peroxidase (*per*) transcript expression was significant (p <0.05) in almost all treatments with mature leaf treatments (Fig 12E, S7 Table). The *per* increment was 3.9 and 9 times higher for IW and CW, respectively, after 3h of exposure to the wind, in relation to the control. In young leaves, there was a significant reduction (p<0.05) in the expression of this transcript when exposed to IW and a significant increase (p<0.05) of up to 0.8 times more, parallel to the exposure times (Fig 12F). Regarding the expression of the *Cu-Zn-sod* in the chloroplast, there was reduction for most treatments for young and mature leaves (Fig 12G and 12H, S7 Table). However, the expression of *Cu-Zn-SOD* in the cytoplasm increased significantly (p<0.05) 2.5 times more in T2 in the mature leaves exposed to CW. In addition, there was a small 1.8 fold increase of this transcript in mature leaves exposed to IW (Fig 12I). In young leaves, the increment was only 0.6 and 0.2 times in T1 and T3 for CW, respectively (Fig 12J, S7 Table).

### Multivariate analysis

Multivariate analysis were performed to determine, separately, which leaf gas exchange and antioxidant metabolism variables presented, in each distinct group, the largest contribution in the evaluation of mechanic stress tolerance.

**Fig 13:**
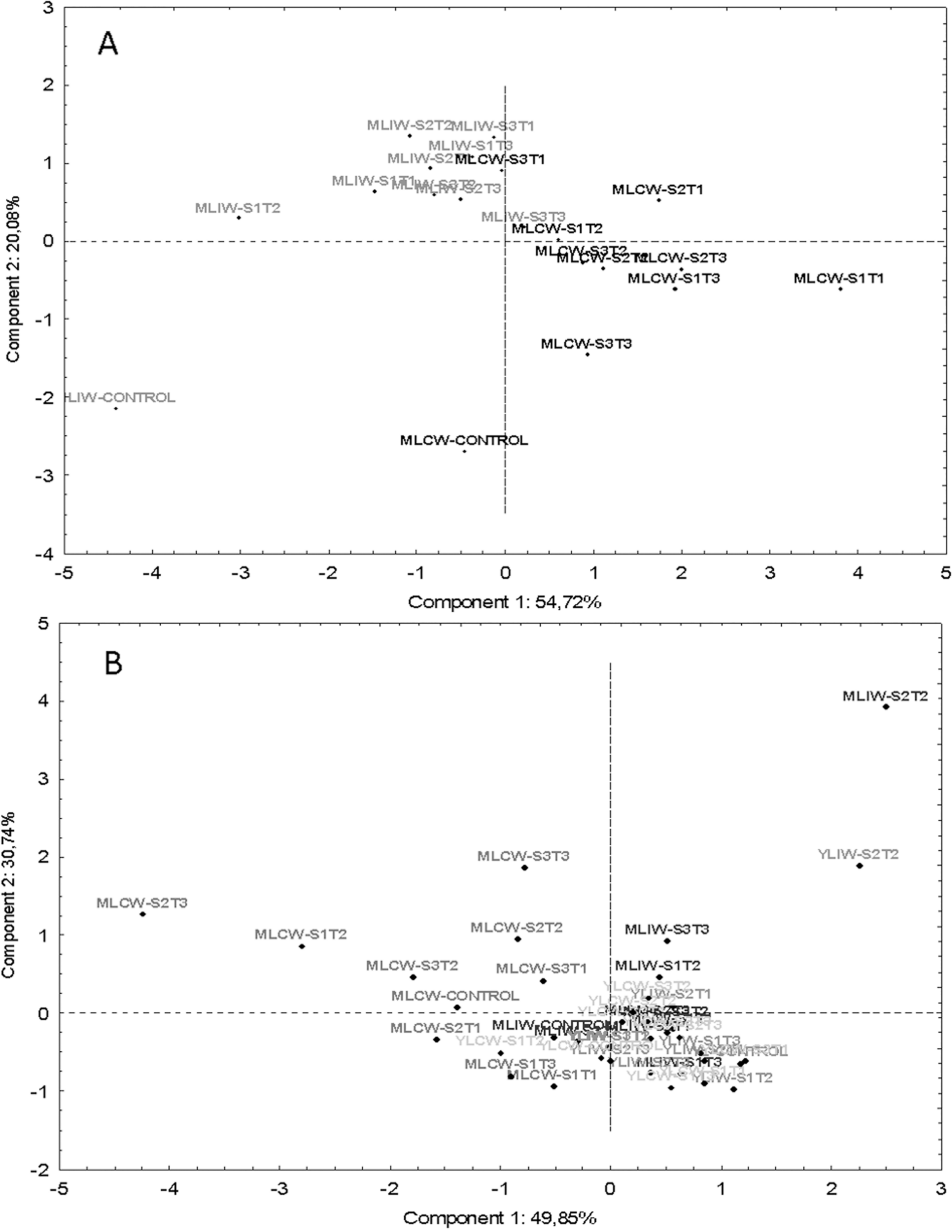
Principal components analysis for leaf gas exchange (A) and biochemical (B) traits of CCN 51 cloned plants of *T*. *cacao* submitted to mechanical stress promoted by wind action.

We found that the leaf gas exchange parameters (*E*, *gs* and *A/gs*) showed greater contribution to the formation of the first component (Fig 13A), whereas the variables (*A* and *Ci*) contributed to the formation of the second component. The first and second component of the leaf gas exchange variables group aproximately 55% and 20%, respectively, of the total variance, with an accumulated autovalue of 75% (Table 2). The physiological variables of the first component formed two groups, according to the imposition of mechanic stress (intermittent and constant wind). The MLIW-Control and MLIW-S1T2 plants that positioned in the negative side of the axis showed significant *gs* and *E* increase. On the other hand, MLCW-S1T1 and MLCW-S2T1 plants, that positioned in the positive side of the axis showed significant increase *A/gs* and decrease of gs and *E* in comparison to the plants submitted to stress. Based on the second component, the leaf gas exchange variables *A* and *Ci* showed the greatest contributions, separating the combinations of intermittent and constant wind conditions. The MLCW-Control and MLIW-Control that positioned in the negative side of the axis showed significant increased *A* in comparison with the plants submitted to stress (Fig 13A).

**Table 2:** Principal components obtained from the correlation matrix of physiological traits CCN 51 cloned plants leaves of *T*. *cacao* submitted to the mechanical stress promoted by wind action.

Variables	PC 1	PC 2
*A*	-	0.78
*gs*	-0.92	-
*E*	-0.96	
*VPDL*	-0.39	-
*A/gs*	0.83	-
*Ci*	0.64	-
Eigenvalue	3.28	1.21
% total variance	54.7	20.0
Cumulative %	54.7	74.8

Biochemical variables with higher contribution in the formation of the first component were GPX and APX, while the TBARS variable contributed to the formation of the second component (Fig 13B). The first and second components, of the biochemical variables, aproximately 50% and 31%, respectively, of the total variance, with an accumulated autovalue of 81% (Table 3). Based on the first component, for biochemical variables, the MLCW-S1T2 and MLCW-S2T3 plants showed larger values of APX and GPX, respectively. On the other hand, based on the second component, the treatment plants MLIW-S2T2 was located on the upper axis of the graph, due to the larger values of TBARS (Fig 13B).

**Table 3:** Principal components obtained from the correlation matrix of biochemical traits CCN 51 cloned plants leaves of *T*. *cacao* submitted to the mechanical stress promoted by wind action.

Variables	PC1	PC2
GPX	-0.80	-
MDA	-	0.89
APX	-0.81	-
Eigenvalue	1.50	0.92
%total variance	49.9	30.7
Cumulative %	49.9	80.6

## Discussion

### The wind promoted macroscopic and ultrastructural changes

Although there were macroscopic and ultrastructural injuries, especially in young leaves exposed to CW, there was no fall of leaves in the CCN 51 cloned plants. Previous experiments with *T*. *cacao* that evaluated the influence of solar and wind radiation also showed macroscopic and microscopic rupture of the foliar tissues [30]. According to these authors, the visible damage to the pulvinus occurred in a short time after exposure to wind (2.4 m s^-1^) and the injury progressed rapidly, causing intense leaf fall and severe damage to the vascular tissues, the epidermis and the cortex. This, in turn, shows that there is a difference, in relation to wind intolerance, between leaves originating from cloned and seminal plants. In the present work, clonal plants wereobtained from productive plagiotropic branches of 10-year-old plants, whose leaves maintain the physiological characteristics of adult plants, unlike young seminal plants, which have juvenile orthotropic leaves.

The disruption of cell walls and of cellular membranes of leaf pulvinus from the CCN 51 cloned plants exposed to CW were more evident in relation to IW, especially in young leaves (Fig 3). These, in turn, have little stiffness in the cell wall, because they are in the process of development. The cell wall is a complex structure that fulfills a variety of functions throughout the life cycle of the plant. In addition to maintaining structural integrity by resisting internal hydrostatic pressures, the cell wall provides flexibility to support cell division and acts as a barrier against environmental and pathological interference in defense against stresses [3]. Plant species of *Festuca arundinacea* [31] and *Acer pseudoplatanus* L. seedlings [32] when submitted to wind showed rupture in the cell wall causing damage to the epidermis, in addition to other types of biotic and abiotic stress that provoked changes in the cell wall [33,34]. The greatest damages occurred in the leaf pulvinus, which can be due to the fact that this basal leaf structure is responsible for the leaf movements and receives the impact of its movement. In addition to the impacts on the cell wall of the young leaflet and the PCD (Fig 5B), the wind caused the disintegration of the mitochondrial crests of the mesophyll cells (Fig 4C), which may have been caused by excess ROS, since mitochondria are an important source of free radicals [35]. In addition, disintegration of cellular organelles can occur after degradation of the nucleus and PCD in plants [36].

Another class of cell death that can occur in plants is vacuolar PCD [36]. Cell death is preceded by the appearance of small vacuoles in the cytoplasm that fuse to form a large vacuole [36]. In this case, the cytoplasm thus becomes replaced by the vacuolar volume. A considerable number of cytoplasmic organelles, in particular plastids, ribosomes, endoplasmic reticulum and peroxisomes disappear during this process [36,37]. In addition, in the final step of the execution of vacuolar PCD, there is rupture of tonoplast and massive release of vacuolar hydrolases [37]. Interestingly, in the present work we have observed the appearance of large lithic vacuoles very similar to those described by [37], as well as the nuclear retraction caused by the increase of the vacuole (Fig 3E). Other studies have also shown that the presence of vesicles and also the chromatin condensation (Fig 4F) are both strong indication of PCD [38–40]. In the present study, the formation of vesicles was observed in young leaf petioles exposed to CW for 12 h (Fig 4E and 4F). The detection of PCD in the petioles, performed *in situ* by the TUNEL technique in this work, is in agreement with other studies that relate the formation of lithic vacuoles and the appearance of vesicles as one of the stages of PCD [34–37].

### The wind altered leaf gas exchanges

The reduction in *A* values in leaves of the CCN 51 cloned plants, when exposed to IW (Fig 6A), is in agreement with the results found in other plant species, such as *Sinapis alba L*. [41], *Pyrus malus L* [15] and *Hibiscus sp* [9] when exposed to wind. According to this author [6], this decrease can be caused by the reduction in the thickness of the boundary air layer on the leaf surface. The reduction of the boundary air layer is determined by the wind speed. In this case *gs* largely controls the leaf water loss [10]. Partial reduction of the boundary layer resistance may promote an increase in *A* as a function of the increases of *gs* and *E*, whereas its total elimination from the blade surface may interfere with leaf temperature and promote stomata closure and compromise the photosynthetic activity. Previous work has already shown that the mechanical stimulus, caused by the wind action, can cause physiological changes in the plants and compromise their development and productivity [7].

In this experiment, the values of *gs* decreased significantly (p<0.05) when the leaves of the CCN51 cloned plants were exposed to IW and CW (Fig 6C and 6D). The reduction of *gs* and consequently of *A*, occurred in detriment to the partial closure of the stomata and therewith, of the reduction of CO_2_ entry through the stomata. Similar results were found in *Malus domestica* plants [15]. In addition, with the closure of stomata, there was also a reduction in *E* values (Fig 6E and 6F) and in *Ci* values (Fig 6G and 6H), for most IW and CW treatments.

The stomatal aperture influences the values of E and A and the internal water balance and many environmental factors influence the stomatal aperure. Wind conditions, especially during the dry season of the year, markedly reduce the production of *T*. *cacao* beans, causing excessive loss of water or reducing leaf area [4] In part, this is due to the reduction of relative air humidity (RH) caused by wind. Direct responses of stomata to RH have been reported by different authors [42, 43]. *T*. *cacao* plants grown under different RH conditions showed significantly different rates of A, E, gs and A/E [43]. According to these authors, A values were consistently higher in conditions of high than in low RH. In this case, *T*. *cacao* plants used water more efficiently at high RH conditions than at low, reflected both by lower values of E and higher than A in conditions of high RH. The extreme sensitivity of *T*. *cacao* plants to low RH may be a limiting factor for the growth of this species, especially in areas with low RH and high wind speeds. In such areas, the growth of *T*. *cacao* plants will be adversely affected by stomatal closure and A reduction. In addition, low A/E values in RH low would lead to higher water deficits under conditions of limited soil water supply.

Also, a increase (p<0.05) in WUE values with increasing speed of IW and time of exposure to wind, in relation to the control, in all treatments combinations (Fig 8A), thus improved plants’ ability to conserve water during photosynthesis. This was also observed by this author [9] when subjecting *Acer pseudoplatanus* to wind. On the other hand, leaves of the CCN 51 cloned plants exposed to CW showed a reduction in WUE values in the S3T2 combinaton (Fig 8B), due to the reduction of gs and, consequently, of E and Ci. Similar results on WUE reduction were also found in *Hibiscus* sp [9], *Pachira macrocarpa* and *Messerschmidia argentea* [8] when exposed to wind.

### Wind increased the expression of psba and reduced that of psbo

When the mature leaves of the CCN51 cloned plants were exposed to IW, with rates of 4.5 m s^-1^, there was an increase in *psba* gene expression that was proportional to the exposure time (Fig 12A). In contrast, young leaves exposed to the same type of wind presented different results than observed in mature leaves. Young leaves showed a decrease in *psba* expression. The *psba* gene encodes for the PsbA protein or D1 protein, which form part of the complex core of the photosystem 2 (PS2) reaction center of the photochemical phase of photosynthesis. The PsbA protein provides binders for cofactors and inorganic ions that catalyze oxidation of the water molecule and transfer electrons to PS2 during the photochemical phase of photosynthesis [44].

The increase in *psba* expression in mature leaves may have occurred in order to increase PS2 activity to compensate for the reduction of photosynthesis (*A)*. Several studies on plants and cyanobacteria have found similar results [24,45,46]. The decrease in *psba* expression in young leaves may have occurred due to oxidative stress caused by the wind. Several studies have demonstrated that plant damage under stress can be triggered by ROS, whose most critical damage occurs at the level of the PS2 protein D1 [45,47,48]. On the other hand, ROS production in turn plays a key role in inducing the production of molecular chaperones. Certain chaperones are related to the plant defense system, acting both on hypersensitivity responses and on acquired systemic resistance [49].

Wind also caused a decrease in *psbo* expression, both in young and in mature leaves (Fig 12C and 12D). The *psbo* gene encodes a PsbO membrane protein, located in the PS2 reaction center, considered to be the protein responsible for the oxidation of the water molecule. This protein is considered essential for the efficient and stable evolution of oxygen, as well as acting as a limiting factor for photosynthesis and growth and interfering with the concentration of other proteins, such as PsbA [50]. Artificial mutants of *Arabidopsis thaliana* are characterized by two genes expressing two PsbO proteins (PsbO-1 and PsbO-2), which are related to the efficiency of the photochemical phase of PS2 and to the D1 protein of the reaction center of PS2 [51]. A regulatory protein may repress gene expression by binding to the control sequences or by playing a positive or supporting role in activating or enhancing the expression of its target gene [52]. Due to the important metabolic roles of PsbO and PsbA, the occurrence of defects in such proteins leads to the phenomenon observed in natural mutants of *T*. *cacao* from the Parinari series, which was characterized by a block in the photosynthetic electron transport chain, leading to the inhibition of photosynthesis [53].

### Wind induced lipid peroxidation and oxidative stress

In general, the amount of TBARS shows the level of lipid peroxidation in cell membranes of plant tissues, when plants are exposed to different environmental stresses [54]. The TBARS accumulation occurred in young and mature leaves when submitted to IW and CW (Fig 9A and 9B). Lipid peroxidation is one of the main effects caused by the increase of ROS. Most cell compartments in higher plants have the potential to become a source of ROS, mainly chloroplasts and mitochondria. Environmental stresses, such as excessive wind speed, which limit CO_2_ fixation, reduce NADP^+^ regeneration by the Calvin cycle, consequently the transport chain of photosynthetic electrons is reduced, producing radicals such as O_2_^-^, H_2_O_2_, and OH^-^ in chloroplasts [55–57]. Increased ROS concentration, when not eliminated by enzymatic and non-enzymatic systems, causes oxidative damage to membranes, proteins, RNA and DNA molecules and may even lead to cell destruction through oxidative stress [58,59]. This fact was evidenced in the present work. The increase of TBARS promoted oxidative stress in leaves of plants exposed to CW, once there was rupture of cell membranes and programmed cell death (Fig 5A and 5B).

Antioxidant enzymes play a key role in the removal of ROS and their activation is directly related to defense against abiotic stress and in plant development [60]. In the present work, the accumulation of ROS induced increases in GPX activity in young and mature leaves exposed to CW, in the S3T2 and S1T2 combination, respectively (Fig 10D and 10C). In these same leaves, APX activity also increased in S2T3 combination (Fig 11C). The induction of defense-related peroxidase activity (GPX and APX) in plants occurs in response to many biotic and abiotic stimuli [58,61,62]. Peroxidases are enzymes that simultaneously catalyze substrate oxidation and H_2_O_2_ reduction, and act on several essential metabolic processes, including regulation of cell growth [63], lignification, phenolic oxidation, defense against pathogens, and protection [64].

Several studies have already shown that acclimatization of plants to environmental changes begins with the molecular perception of stress. In addition, the signal transduction occurs by seeking the regulation of genes, which may undergo large variations in their expression induced by stress [65,66]. Changes in the expression of certain gene-encoding antioxidant enzymes have been observed. Increased expression of *per* transcripts was significant in mature leaves at all CW and IW exposure times (Fig 12E). The highest increment was found in mature leaves, after 3 h of exposure to CW. These results confirm the activity of GPX in mature and young leaves exposed to CW and IW, except in mature leaves exposed to IW. However, in young leaves, the increment in these transcripts was insignificant, especially for the leaves exposed to IW (Fig 12F), whereas the GPX enzymatic activity for these treatments was significant.

The expression of *Cu-Zn-sod* in the chloroplast decreased or remained constant for all treatments (Fig 12G and 12H). The *Cu-Zn-sod* enzyme disassembles the superoxides produced in the chloroplasts. The production of ^-1^O_2_ can cause reprogramming of the expression of other genes, leading to chlorosis and programmed cell death, as well as induce a wide range of responses related to biotic and abiotic stress [67,68]. The non-alteration of *Cu-Zn-sod* transcripts can be explained by the integrity of the chloroplasts observed in the structural photomicrographs (Fig 4). In the present work, has been shown that high concentrations of H_2_O_2_ under abiotic stress can be a result of the action of SOD in superoxide radicals and the direct formation of H_2_O_2_ in biochemical pathways such as photorespiration [69,70]. The *Cu-Zn-sod* expression in the cytoplasm of young leaf tissue cells, increase only in the period of 3 h in CW (Fig 12J).There was an increase of this transcript in mature leaves exposed to IW, at 3, 6 and 12 h. However, the highest increase in expression of this gene was in mature leaves exposed to CW (Fig 12I). Previous work has already demonstrated that post-transcriptional induction of *Cu-Zn-sod* genes in *Arabidopsis* is critical for oxidative stress tolerance [71]. The increase of *Cu-Zn-sod* transcripts may have contributed to the increase in the tolerance of mature leaves of CCN 51 to the mechanical stress caused by the wind.

## Conclusions

The young and mature leaves of the CCN 51 cloned plants of *T*. *cacao* presented differential responses when exposed to intermittent (IW) and constant (CW) wind. The young leaves were more intolerant to the mechanical stress caused by the wind, since they presented greater macro and microscopic damages and, consequently, greater molecular, biochemical and ultrastructural changes.

The pulvinus and the lamina of mature and young leaves, exposed to IW and CW with different exposure times (3 and 12 h), showed notable macroscopic and microscopic mechanical injuries, resulting from constant leaf movement. At both speeds there were ultrastructural alterations, involving rupture of the cell membranes of the pulvinus and mesophyll tissues, followed by programmed cell death, mainly in the tissues of young leaves.

It was verified that CW and IW, with different exposure times, interfered directly in stomatal conductance (*gs*), transpiration (*E*) and water use efficiency (*WUE*), causing a reduction of the photosynthetic rate (*A*) in mature leaves.

The action of CW and IW, at different speeds and exposure times on young and mature leaves, promoted oxidative stress, and consequently, increased activity of guaiacol peroxidase (GPX) and ascorbate peroxidase (APX) for most treatments. In addition, there was alteration in the expression of transcripts of the *psba* and *psbo* and the *Cu-Zn-sod* and *per* genes related to the antioxidative enzymes at the rate of 4.5 ms^-1^.

Therefore, high wind speed can seriously compromise the development of young leaves of *T*. *cacao* plants and affect their productivity.

The results demonstrated the need for the use of peripheral protection barriers in adult cocoa plantations, providing lateral shading and protection against excessive winds, rather than the use of conventional top shading. On the other hand, some cacao producing regions in the full sun face serious problems with the mechanical effects caused by the wind. It would be interesting to use trees of greater economic value in peripheral barriers of protection against the wind, since it will add greater economic value to the production of cocoa. Besides that, cocoa cultivation in full sun, in addition to increasing production about 10 times more than the shade cultivation, makes possible the prophylactic control of fungal diseases, such as witches' broom and brown rot, which has *Moniliophthora perniciosa* and *Phytophthora* sp as causal agents, respectively, as a result of microclimatic changes that lead to lower relative humidity.

## Supporting information

S1 Table Numerical data. Net photosynthesis (A), stomatal conductance (gs), transpiration (E), internal CO_2_ concentration in leaf mesophyll (Ci) and water steam pressure deficit between leaf and air (VPDL) in CCN 51 cloned plants of *T*. *cacao* exposed to intermittent (IW) and constant (CW) wind at different speeds (2.5, 3.5 and 4.5 m s^-1^) and exposure times (0, 3, 6 and 12 h).
(XLSX)

S2 Table Numerical data. Relationship between net photosynthesis (*A*) and stomatal conductance (*gs*) in CCN 51 cloned plants of *T*. *cacao* exposed to intermittent and constant wind at different speeds [2.5 (S1), 3.5 (S2) and 4.5 (S3) m s-1] and exposure times [3 (T1), 6 (T2) and 12 (T3) h)—S1T1, S1T2, S1T3, S2T1, S2T2, S2T3, S3T1, S3T2 and S3T3.
(XLSX)

S3 Table Numerical data. Instant water use efficiency (WUE) on leaves of CCN 51 cloned plants of *T*. *cacao exposed to* intermittent and constant winds with different speeds [2.5 (S1), 3.5 (S2) and 4.5 (S3) m s^-1^] and exposure times [0 (C), 3 (T1), 6 (T2) and 12 (T3) h).
(XLSX)

S4 Table Numerical data. Concentration of thiobarbituric acid-reactive substances (TBARS) in leaves of CCN 51 cloned genotype of *T*. *cacao* exposed to intermittent (IW) and constant (CW) wind with different speeds (2.5, 3.5 and 4.5 m s^-1^) and exposure times (0, 3, 6 and 12 h).
(XLSX)

S5 Table Numerical data. Activity of guaiacol peroxidase (GPX) in leaves of CCN 51 cloned plants of *T*. *cacao* exposed to wind at different speeds (2.5, 3.5 and 4.5 m s-1) and exposure times (0, 3, 6 and 12 h).
(XLSX)

S6 Table Numerical data. Activity of ascorbate peroxidase (APX) in leaves of CCN 51 cloned plants of *T*. *cacao* exposed to wind at different speeds (2.5, 3.5 and 4.5 m s^-1^) and exposure times (0, 3, 6 and 12 h).
(XLSX)

S7 Table Numerical data. Relative expression of *psba*, *psbo*, *per*, *Cu-Zn-sod* in the chloroplast and cytoplasm genes in young and mature leaves of CCN 51 cloned plantas of *T*. *cacao* exposed to intermittent and constant wind.
(XLSX)

## References

[1] Coutand C. Mechanosensing and thigmomorphogenesis, a physiological and biomechanical point of view. Plant Science. 2010; 79(3): 168–182.

[2] Puijalon S, Bouma TJ, Douady CJ, Groenendael JV, Niels PR, Anten EM, et al Plant resistance to mechanical stress: evidence of an avoidance–tolerance trade-off. New Phytologist. 2011; 191: 1141–1149. doi: 10.1111/j.1469-8137.2011.03763.x 21585390 10.1111/j.1469-8137.2011.03763.x

[3] Houston K, Tucker MR, Chowdhury J, Shirley N, Little A. The plant cell wall: a complex and dynamic structure as revealed by the responses of genes under stress conditions. Frontiers in Plant Science. 2016; 10(7): 984. 10.3389/fpls.2016.00984 PMC4978735 27559336

[4] Alvim PT, Alvim R and Leite RM. Mechanical injury of wind to recently transplanted cacao. Seedlings as related to the shade problems. Revista Theobroma, Brasil. 1978, 8(4): 117–124.

[5] Braam J. In touch: plant responses to mechanical stimuli. New Phytologist. 2005; 165(2): 373–389. doi: 10.1111/j.1469-8137.2004.01263.x 15720650 10.1111/j.1469-8137.2004.01263.x

[6] Wada H, Masumoto-Kubo C, Gholipour Y, Nonami H, Tanaka F, et al Rice chalky ring formation caused by temporal reduction in starch biosynthesis during osmotic adjustment under foehn-induced dry wind. PLoS ONE. 2014; 9(10): e110374 doi: 10.1371/journal.pone.0110374 25330305 10.1371/journal.pone.0110374 PMC4203794

[7] Langre E. Effects of Wind on Plant. The Annual Review of Fluid Mechanicsis. 2008; 40: 141–68.

[8] Huang CW, Chu CR, Hsieh CI, Palmroth S, Katul GG. Wind-induced leaf transpiration, Advances in Water Resources. 2016; 86: 240–255.

[9] Schymanski JS and Or D. Wind increases leaf water use efficiency. Plant, Cell and Environment. 2016; 39: 1448–1459. doi: 10.1111/pce.12700 26714739 10.1111/pce.12700

[10] Taiz L and Zeiger E. Plant Physiology. 2013; 5ed Artmed.

[11] Onoda Y and Anten NPR.Challenges to understand plant responses to wind. Plant Signaling & Behavior.2011; 6 (7): 1057–1059. 21617382 10.4161/psb.6.7.15635 PMC3257795

[12] Nagano S, Nakano T, Hikosaka K and Maruta E. Needle traits of an evergreen, coniferous shrub growing at wind-exposed and protected sites in a mountain region: does *Pinus pumila* produce needles with greater mass per area under wind-stress conditions? Plant Biology. 2009; 1435: 8603. 10.1111/j.1438-8677.2009.00253.x 19778373

[13] Lambers H, Chapin III FS and Pons TL. Photosynthesis, respiration, and long-distance transport In Plant physiological ecology. Springer New York 1998; 10–153.

[14] Ennos AR. Wind as an ecological factor. Trends in Ecology and Evolution. 1997; 12: 108–111. 21237994 10.1016/s0169-5347(96)10066-5

[15] Tartachnyk I, Michael MB. Effect of mechanically simulated hail on photosynthesis, dark respiration and transpiration of apple leaves. Environmental and Experimental Botany. 2002; 48: 169–175.

[16] Choudhury FK, Rivero RM, Blumwald E and Mittler R. Reactive oxygen species, abiotic stress and stress combination. The Plant Journal. 2016; 90(5): 856–867. doi: 10.1111/tpj.13299 27801967 10.1111/tpj.13299

[17] Choudhury S, Panda P, Sahoo L, Panda SK. Reactive oxygen species signaling in plants under abiotic stress. Plant Signaling & Behavior. 2013; 8(4): e23681. 23425848 10.4161/psb.23681 PMC7030282

[18] Foyer CH and Noctor G. Redox signaling in plants. Antioxidants Redox Signaling. 2013; 18: 2087–2090. doi: 10.1089/ars.2013.5278 23442120 10.1089/ars.2013.5278

[19] Considine MJ, Sandalio LM. and Foyer CH. Unravelling how plants benefit from ROS and NO reactions, while resisting oxidative stress. Annals Botany. 2015; 116: 469–473. 10.1093/aob/mcv153 PMC4578007 26649372

[20] Dietz KJ. Efficient high light acclimation involves rapid processes at multiple mechanistic levels. Journal Experimental Botany. 2015; 66: 2401–2414. 10.1093/jxb/eru505 25573858

[21] Mignolet-Spruyt L, Xu E, Idanheimo N, Hoeberichts FA, Muhlenbock P, Brosche M, et al Spreading the news: subcellular and organellar reactive oxygen species production and signalling. Journal Experimental Botany. 2016; 67: 3831–3844. 10.1093/jxb/erw080 26976816

[22] Schmutz J, Cannon SB, Schlueter J, Ma J, Mitros T, Nelson W, et al Genome sequence of the palaeopolyploid soybean. Nature. 2010; 463: 178–183. doi: 10.1038/nature08670 20075913 10.1038/nature08670

[23] Toth SZ, Nagy V., Puthur JT, Kovacs L, Garab G. The physiological role of ascorbate as photosystem II electron donor: protection against photo inactivation in heat-stressed leaves. Plant Physiology. 2011; 156: 382–392. doi: 10.1104/pp.110.171918 21357184 10.1104/pp.110.171918 PMC3091034

[24] Gururani MA, Venkatesh J, Tran LSP. Regulation of Photosynthesis during Abiotic Stress-Induced Photoinhibition. Review. Molecular Plant. 2015; 8 (9): 1304–1320. doi: 10.1016/j.molp.2015.05.005 25997389 10.1016/j.molp.2015.05.005

[25] Bertolde FZ, Almeida A-A.F, Pirovani CP, Gomes FP, Ahnert D, Baligar VC and Valle RR. Physiological and biochemical responses of Theobroma cacao L. genotypes to flooding. Photosynthetica. 2012; 50 (3): 447–457.

[26] Kar M and Mishra D. Catalase, peroxidase, and polyphenoloxidase activities during rice leaf senencence. Plant Physiology. 1976; 57(2):315–319. 16659474 10.1104/pp.57.2.315 PMC542015

[27] Koshiba T. Cytosolic ascorbate peroxidase in seedlings and leaves of maize (*Zea mays*). Plant Cell Physiology. 1993; 34(5):713–721.

[28] Heath RL, Packer L. Photoperoxidation in isolated chloroplasts. I. Kinetics and stoichiometry of fatty acid peroxidation. Archives in Biochemistry and Biophysics. 1968; 125:189–198. 10.1016/0003-9861(68)90654-1 5655425

[29] Livak KJ, Schmittgen TD. Analysis of relative gene expression data using real-time quantitative PCR and the 2-DDC T method Methods. 2001; 25: 402–408. 10.1006/meth.2001.1262 11846609

[30] Leite RMO. Efeito da ação do vento e da radiação solar na ruptura do pulvínulo foliar do cacaueiro (*Theobroma cacao L*.).1978; (Tese).

[31] Thompson JR. The ffect of wind on grasses. II. Mechanical damage in *Festuca arundi-nacea* Schreb. Journal Experimental Botany. 1974; 25: 965–972.

[32] Wilson J. Microscopic features of wind damage to leaves of *Acer pseudoplatanus* L. Annals of Botany.1984 53: 73–82.

[33] Caño‐Delgado A, Penfield S, Smith C, Catley M, Bevan M. Reduced cellulose synthesis invokes lignification and defense responses in *Arabidopsis thaliana*. Plant Journal. 2003; 34(3): 351–362. 12713541 10.1046/j.1365-313x.2003.01729.x

[34] Bellin campi D, Cervone F, Lionetti V. Plant cell wall dynamics and wall-related susceptibility in plant–pathogen interactions. Frontiers Plant Science. 2014; 5: 228. 10.3389/fpls.2014.00228 PMC4036129 24904623

[35] Salin ML.Chloroplast and mitochondrial mechanisms for protection against oxygen toxicity. Free Radical Research Communications. 1991; 12(13): 851–858. 2060857 10.3109/10715769109145867

[36] Fukuda H. Programmed cell death of tracheary elements as a paradigm in plants, Plant Molecular Biology. 2000; 44: 245–253. 11199386 10.1023/a:1026532223173

[37] Van Doorn WG. Classes of programmed cell death in plants, compared to those in animals. Journal Experimental Botany. 2011; 62: 4749–61. 10.1093/jxb/err196 21778180

[38] Evans DE. Aerenchyma formation. New Phytologist. 2004 161(1):35–49.

[39] Dauphinee AN, Warner TS, Gunawardena AH. A comparison of induced and developmental cell death morphologies in lace plant (*Aponogeton madagascariensis*) leaves. BMC plant biology. 2014; 14(1): 389. 25547402 10.1186/s12870-014-0389-x PMC4302576

[40] Papini A, Sordo L, Mosti S. Interacções superficiais da macroalga epifítica *Hincksia mitchelliae* (*Phaeophyceae*) com o capim-bravo, *Halodule wrightii* (*Cymodoceaceae*). Journal of Phycology. 2011; 47 (1): 118–122. doi: 10.1111/j.1529-8817.2010.00935.x 27021717 10.1111/j.1529-8817.2010.00935.x

[41] Retuerto R, Woodward FI. The influences of increased CO_2_ and water supply on growth, biomass allocation and water use efficiency of *Sinapis alba L*. grown under different wind speeds. Oecologia. 1993; 94: 415–427. doi: 10.1007/BF00317118 28313680 10.1007/BF00317118

[42] Kozlowski TT and Pallardy SG. Effects of flooding on water, carbohydrate, and mineral relations In: Flooding and Plant Growth. Academic Press 1984; 165–193.

[43] Gomes SAR, Kozlowski TT, Reich PB. Some physiological responses of Theobroma cacao var. catongo seedlings to air humidity. New Phytologist. 1987; 107:591–602.

[44] Nelson N and Yocum CF. Structure and function of photosystems I and II. Annual Review of Plant Biology. 2006; 57: 521–565. doi: 10.1146/annurev.arplant.57.032905.105350 16669773 10.1146/annurev.arplant.57.032905.105350

[45] Souza VL, Almeida A-AF, Souza SJ, Mangabeira OPA, Jesus RM, Pirovani C P, Ahnert D, Baligar VC, et al Altered physiology, cell structure, and gene expression of *Theobroma cacao* seedlings subjected to Cu toxicity. Environmental Science and Pollution Research. 2013; 21 (2): 1217–1230. doi: 10.1007/s11356-013-1983-4 23888348 10.1007/s11356-013-1983-4

[46] Santos IC, Almeida A-AF, Anhert D, Conceição AS, Pirovani CP, Pires JL, et al Molecular, physiological and Bbiochemical responses of *Theobroma cacao* L. genotypes to soil water deficit. PLoS ONE. 2014; 9(12): e115746 doi: 10.1371/journal.pone.0115746 25541723 10.1371/journal.pone.0115746 PMC4277404

[47] Yamamoto Y, Aminaka R, Yoshioka M, et al Quality control of photosystem II: impact of light and heat stresses. Photosynth Research. 2008; 98:589. 10.1007/s11120-008-9372-4 18937045

[48] Calderone V, Trabucco M, Vujicić A, Battistutta R, Giacometti GM, Andreucci F, Barbato R, Zanotti G. Crystal structure of the PsbQ protein of photosystem II from higher plants. EMBO Reports. 2003: 4(9): 900–905. doi: 10.1038/sj.embor.embor923 12949587 10.1038/sj.embor.embor923 PMC1326360

[49] Lipka V and Panstruga R. "Dynamic cellular responses in plant–microbe interactions." Current opinion in plant biology. 2005; 8(60): 625–631. 16182598 10.1016/j.pbi.2005.09.006

[50] Murakami R, Ifuku K, Takabayashi A, Shikanai T, et al Functional dissection of two *Arabidopsis* PsbO proteins: PsbO1 and PsbO2. FEBS Journals. 2005; 272: 2165–2175. 10.1111/j.1742-4658.2005.04636.x 15853801

[51] Liu Y, Schieving F, Stuefer JF, Anten NP. The effects of mechanical stress and spectral shading on the growth and allocation of ten genotypes of a stoloniferous plant. Annals Botany. 2007; 99: 121–130. 10.1093/aob/mcl230 PMC2802973 17085473

[52] Brown TA. Clonagem Gênica e Análise de DNA: Uma introdução 4ª ed ArtMed, 2003.

[53] Rehem BC, Almeida A.-AF, Santos IC, Gomes FP, Pirovani CP, Mangabeira PAO, Corrêa RX, Yamada MM, Valle RR. Photosynthesis, chloroplast ultrastructure, chemical composition and oxidative stress in *Theobroma cacao* hybrids with the lethal gene *Luteus-Pa* mutant. Photosynthetica. 2011; 49 (1): 127–139.

[54] Koca H, Bor M., Zdemir OF, Urkan TI. The effect of salt stress on lipid peroxidation, antioxidative enzymes and proline content of sesame cultivars. Environmental and Experimental Botany. 2007; 60: 344–351.

[55] Wu YS, Tang K.X. MAP Kinase cascades responding to environmental stress in plants. Acta Botanica. 2004; 46:127–136.

[56] Li J, Jin H.Regulation of brassinosteroid signaling. Trends Plant Science. 2007; 12: 37–41. 10.1016/j.tplants.2006.11.002 17142084

[57] Shao HB, Chu LY, Lu ZH, Kang CM. Primary antioxidant free radical scavenging and redox signaling pathways in higher plant cells. International Journal of Biological Sciences. 2008; 4:8–14. 10.7150/ijbs.4.8 PMC2140154 18167531

[58] Mittler R. Oxidative stress, antioxidants and stress tolerance. Trends in Plant Science. 2002; 7 (9): 405–410. 12234732 10.1016/s1360-1385(02)02312-9

[59] Niki E. Lipid peroxidation: Physiological levels and dual biological effects. Free Radical Biology & Medicine, London. 2009; 47: 469–484. 10.1016/j.freeradbiomed.2009.05.032 19500666

[60] Foyer CH and Noctor G. Oxidant and antioxidant signaling in lants: a re-evaluation of the concept of oxidative stress in a physiological context. Plant Cell Environment. 2005; 28: 1056–71.

[61] Teixeira FK, Menezes-Benavente L, Galvão VC, Margis R, Margis-Pinheiro M. Rice ascorbate peroxidase gene family encodes functionally diverse isoforms localized in different subcellular compartments. Planta. 2006; 224: 300–314. doi: 10.1007/s00425-005-0214-8 16397796 10.1007/s00425-005-0214-8

[62] Martinelli F, Reagan RL, Dolan D, Fileccia V and Dandekar AM. Proteomic analysis highlights the role of detoxification pathways in increased tolerance to Huang long bing disease. BMC Plant Biology. 2006; 16:167. 10.1186/s12870-016-0858-5 PMC4963945 27465111

[63] Goldberg R, Imbeerty A, Liberman M, Prat R. Relatioship between peroxidatic activities and cell plasticity In: Greepin H, Penel C, Gasper JT, editors. Molecular and Physiological Aspects of Plant Peroxidases. Switzerland University of Geneva 1986; 208–220.

[64] Lüderitz T and Grisebach H. Enzymic Synthesis of Lignin Precursors Comparison of Cinnamoyl-CoA Reductase and Cinnamyl Alcohol: NADP^+^ Dehydrogenase from Spruce (*Picea abies L*.) and Soybean (*Glycine max L*.). European Journal of Biochemistry. 1981; 119: 115–124. 7042334 10.1111/j.1432-1033.1981.tb05584.x

[65] Kieffer P, Schröder P, Dommes J, Hoffmann L, Renaut J, Hausman J-F. Proteomic and enzymatic response of poplar to cadmium stress. Journal of Proteomics. 2009; 72(3): 379–396. 19367735 10.1016/j.jprot.2009.01.014

[66] Amudha J, Balasubramani G. Recent molecular advances to combat abiotic stress tolerance in crop plants. Biotechnology and Molecular Biology Reviews: 2011; 6(2):31–58.

[67] Wagner D, Przybyla D, Op den Camp R.et al The genetic basis of singlet oxygen-induced stress responses of *Arabidopsis thaliana*. Science. 2004; 306: 1183–1185. doi: 10.1126/science.1103178 15539603 10.1126/science.1103178

[68] Kleine T and Leister D. Retrograde signaling: organelles go networking. Biochimica et Biophysica Acta. 2016; 1857: 1313–1325. doi: 10.1016/j.bbabio.2016.03.017 26997501 10.1016/j.bbabio.2016.03.017

[69] Foyer CH. and Noctor G. Redox regulation in photosynthetic organisms: signaling, acclimation, and practical implications. Antioxidant Redox Signaling. 2009; 11: 861–905. 10.1089/ars.2008.2177 19239350

[70] Sharma P, Jha AB, Dubey RS and Pessarakli M. Reactive Oxygen Species, Oxidative Damage, and Antioxidative Defense Mechanism in Plants under Stressful Conditions. Journal of Botany 2012, 217037: 26.

[71] Sunkar R, Kapoor A, Zhu JK. Posttranscriptional induction of two Cu/Zn superoxide dsmutase genes in *Arabidopsisis* mediated by down regulation of miR398 and important for oxidative stress tolerance. Plant Cell. 2006; 18: 2051–2065. doi: 10.1105/tpc.106.041673 16861386 10.1105/tpc.106.041673 PMC1533975

